# Evaluating neoantigen-vaccine responses through mechanistic and model-based frameworks

**DOI:** 10.1038/s41698-026-01579-8

**Published:** 2026-06-24

**Authors:** Eman I. K. Ibrahim, Ida Laurén, Rosanne E. Veerman, Siv Jönsson, A. Jimmy Ytterberg, Annika Lindqvist, Sara M. Mangsbo, Lena E. Friberg

**Affiliations:** 1 Department of Pharmacy, Uppsala University, Uppsala, Sweden; 2 Department of Pharmacy, Science for Life Laboratory, Uppsala University, Uppsala, Sweden; 3 Strike Pharma AB, Lund, Sweden; 4 Department of Pharmacy, SciLifeLab Drug Discovery and Development Platform, Uppsala University, Uppsala, Sweden

**Keywords:** Cancer, Computational biology and bioinformatics, Drug discovery, Immunology

## Abstract

Therapeutic cancer vaccines activate tumor-specific T-cells to reduce tumor burden, yet face challenges when integrating and translating multi-level complex preclinical data into actionable insights. We present a model-based framework to integrate diverse preclinical data, comparing synthetic long peptide vaccines with or without adjuvant stimuli to a vaccine-drug conjugate approach by Adaptable Drug Affinity Conjugate technology. This technology provides modular and rapid conjugate formation via high-affinity binding between a peptide-tagged vaccine antigen and a CD40-targeting antibody, acting as an adjuvant and delivery vehicle. Our developed semi-mechanistic modelling framework successfully linked dosing to tumor dynamics, incorporating four sub-models: pharmacokinetic, peptide uptake by antigen-presenting cells, T-cell response, and tumor growth in TC-1 and MC38 tumors. Model-based simulations highlighted the importance of affinity conjugation on pharmacokinetics and efficacy. Effector T-cells mediated tumor shrinkage. Antibody dose-dependent effects were identified and quantified in the immune-responsive MC38 model. This framework supports rational vaccine optimization and translational decision-making. The graphical abstract was created in https://BioRender.com.

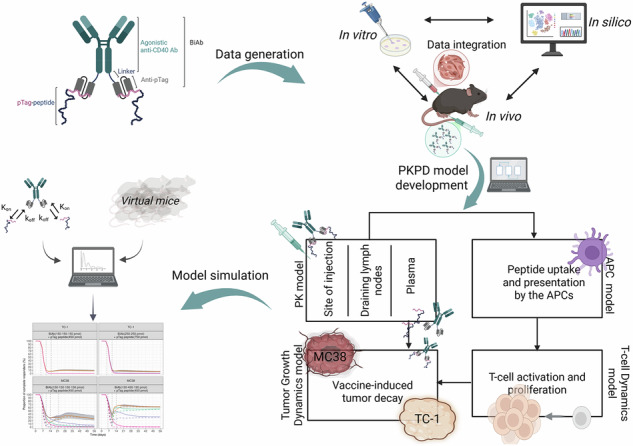

## Introduction

Cancer immunotherapy leverages the immune system’s ability to recognize and eliminate cancer cells by amplifying effector mechanisms and/or blocking inhibitory pathways^1^. Therapeutic cancer vaccines aim to activate tumor-specific T-cells for tumor regression and long-term immunity^2,3^. Despite encouraging preclinical results, clinical success has been limited due to weak effector responses, immune tolerance, and peptide stability challenges^4,5^. Strategies to enhance T-cell responses with peptide-based vaccines have included adjuvant activation of antigen-presenting cells, for example, through co-administration with granulocyte-macrophage colony-stimulating factor (GM-CSF) or CD40 agonists. A central translational question, therefore, remains: what antigen exposure profile, magnitude, duration, and cellular/organ distribution is required to drive an effective T-cell priming and durable anti-tumor effects in vivo? Novel mRNA neoantigen-based therapeutic approaches enable antigen exposure via host neoantigen production following mRNA delivery and thereby providing sustained exposure over time^6^, whereas synthetic long peptide vaccines have a very short half-life, which can be extended by pharmacological design. To better decipher immune system complexity and evaluate how drug design influences tumor response early in development, such approaches can reduce development timelines and improve the likelihood of clinical trial success. These interactive tools also enable visualization of how antigen exposure in relevant organs, such as through targeted drug delivery, shapes vaccine immunogenicity.

Synthetic long peptide (SLP) vaccines have been investigated in preclinical and clinical settings. To enhance exposure and immunogenicity in the clinic, they have been formulated in Montanide-based emulsions or administered following pre-conditioning of the injection site with adjuvants such as GM-CSF or toll-like receptor agonists. However, several Phase III clinical studies that included control arms^7,8^ have failed to demonstrate clinical benefit, opposing the promising results from early phase I/II clinical^9,10^ data and pre-clinical studies in mice^11,12^. One plausible explanation is that the effective antigen exposure at the relevant site(s) of uptake and presentation is highly formulation-dependent and may be insufficient or too transient, particularly for fragile peptide entities susceptible to degradation before reaching antigen-presenting cells (APCs). If so, strategies that stabilize antigen and bias delivery of both antigen and adjuvant to professional APCs could shift the exposure–response relationship and improve the probability of clinical benefit^13,14^.

**Fig. 1:**
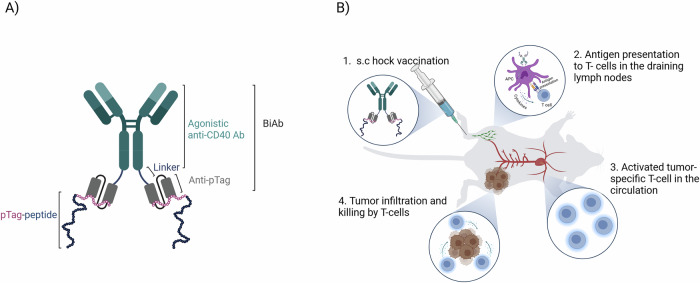
Illustration of the Adaptable Drug Affinity Conjugate (ADAC) platform. Illustration of the Adaptable Drug Affinity Conjugate (ADAC) platform (**A**) and mechanism of action of a bispecific antibody-based cancer vaccine (**B**). This design is called BiTag, consisting of an agonistic anti-CD40 antibody, anti-pTag (i.e., single-chain variable fragments, scFvs), and pTag. This structure can be conjugated to cargo peptides with varying sequences. Created in https://BioRender.com.

Antibody-based delivery has traditionally been advanced through antibody–drug conjugates (ADCs) to direct payloads to defined cell populations^15^. In the cancer vaccination context, the late Ralph Steinman pioneered the work with antibody-targeted vaccination strategies combining DEC205 targeting with CD40 stimulation to boost T-cell activation more efficiently^16^. Building on this strategy, we developed an approach to easily prepare ADC vaccines using the Adaptable Drug Affinity Conjugate (ADAC) platform^17,18^ to facilitate drug loading of synthesized tagged peptide neoantigens by affinity interaction to a tetravalent bispecific antibody (BiAb). This BiAb consists of an agonistic anti-CD40 antibody fused with an anti-peptide Tag (anti-pTag) domain (e.g., single-chain variable fragments, scFvs) through the heavy chain. The high-affinity interaction between the cargo peptide and the BiAb is mediated by the peptide Tag (pTag) sequence, forming a structure referred to as a BiTag (Fig. 1A)^17^. This pTag is designed to enhance peptide stability and targeted delivery to CD40-expressing APCs, enabling personalized cancer vaccines by varying the cargo neoantigen. The drug candidate STRIKE2001, a humanized tetravalent IgG2-based antibody featuring two peptide-binding scFvs linked to the CH3 domain^18^, is under preclinical evaluation and has shown no platelet activation in human blood and no toxicity upon subcutaneous (s.c.) administration in mice^18^. Collectively, these features motivate a key mechanistic hypothesis: prolonging and redirecting antigen exposure via antibody-based delivery can increase the effective antigen presentation, peptide-specific T-cells expansion, and translate to improved tumor control, potentially in a tumor-context–dependent manner.

Testing such hypotheses experimentally across formulations, binding affinities, adjuvant conditions, and tumor immune states is resource-intensive and often underpowered to resolve causality. Model-informed drug development (MIDD) offers a complementary route to integrate multiscale measurements and interrogate mechanisms quantitatively, and is increasingly used to support drug development and regulatory review^19,20^. Crucially, in this setting, the value lies in using biologically grounded models that explain observed outcomes and enable “what-if” simulations to guide formulation and study design. This aligns with emerging concepts of “virtual subject” and “digital twin” for predicting treatment efficacy under specific scenarios^21,22^. Such frameworks can also clarify how conjugated formulations alter pharmacokinetics (PK) and pharmacodynamics (PD), providing insight into the frequent late-stage failure of SLP vaccines.

In this work, we aimed to quantitatively characterize the complex interplay among immune system components and the temporal dynamics underlying the response to SLP vaccines, either formulated in a water-based framework with or without an adjuvant stimulus or delivered via an ADC-based strategy. This objective was achieved by developing a multiscale, semi-mechanistic data-driven pharmacokinetic-pharmacodynamic (PKPD) modeling framework, informed by preclinical data, that captures key processes following ADAC-based product administration, including BiAb:pTag-peptide conjugate PK, peptide uptake, processing, and presentation by APC, in addition to the subsequent peptide-specific T-cell activation and proliferation. To capture the novel product responses across tumor types with differing immunogenicity, the framework was expanded with a PD model describing longitudinal tumor size data from mouse models featuring non-inflamed (TC-1) and inflamed (MC38) tumors across various formulations. The established framework enabled the evaluation of the impact of antigen exposure duration on the magnitude of T-cell and tumor responses, as well as the sensitivity of immune and tumor outcomes to conjugate stability, facilitated by pTag affinity as a key determinant of stability and exposure, through simulations.

## Results

**Fig. 2:**
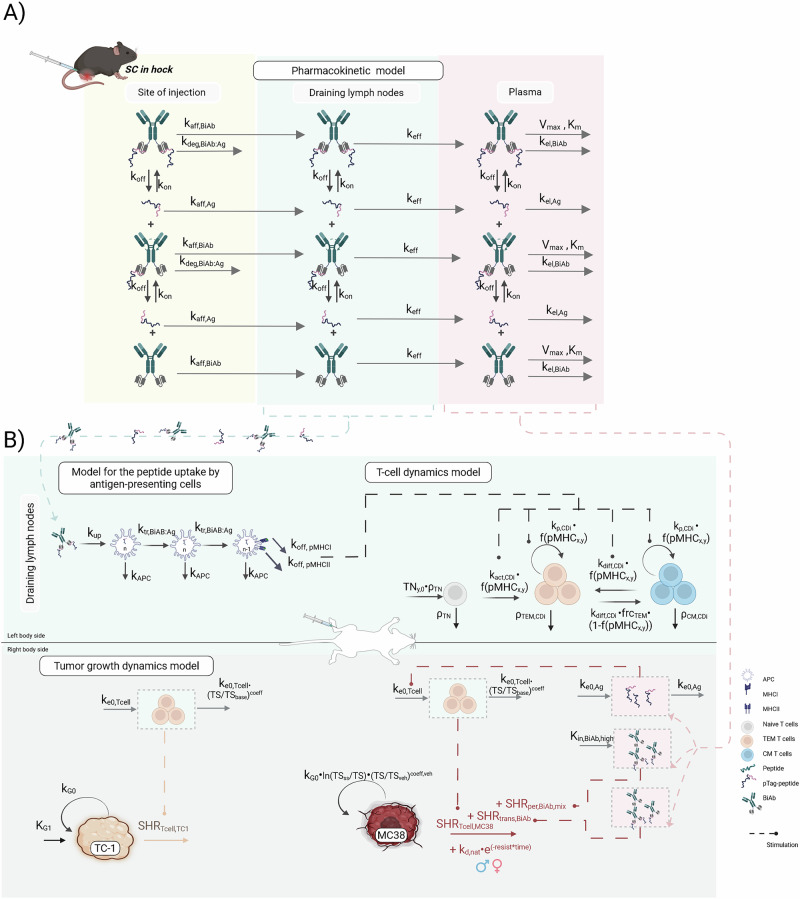
Schematic representation of the multiscale semi-mechanistic pharmacokinetic-pharmacodynamic modeling framework. Schematic representation of the multiscale semi-mechanistic data-driven pharmacokinetic (**A**) -pharmacodynamic (**B**) modeling framework. ${BiAb}$ bispecific antibody, ${Ag}$ peptide, ${APC}$ antigen presenting cell, *pMHC*_*x,y*_ major histocompatibility complex -peptide complexes, *TEM* transition effector memory T-cells; *CM*, central memory T-cells; *k*_*deg*,*BiAb*:*Ag*_, first-order degradation rate constant of BiAb conjugate from injection site, ${{\rm{k}}}_{{\rm{aff}},{\rm{BiAb}}}$ and ${{\rm{k}}}_{{\rm{aff}},{\rm{Ag}}}$ first-order absorption rate constants of *BiAb* and ${Ag}$ into the lymph nodes, respectively; ${k}_{{eff}}$ first-order rate constant of transition from lymph nodes to plasma, ${k}_{{el},{BiAb}}$ and ${k}_{{el},{Ag}}$ first-order elimination rate constants of ${Bi}{Ab}$ and ${Ag}$ from plasma, respectively; ${V}_{\max }$ maximum elimination capacity of total ${Bi}{Ab}$, ${K}_{m\,}$ concentration at which the elimination rate is half-maximal, ${k}_{{up}}$ first-order rate constant of peptide uptake by APC, ${k}_{{tr},{BiAb}:{Ag}}$ first-order rate constant of delayed release of peptide within APC, ${k}_{{APC}}$ first-order turn-over rate constant of APC, ${k}_{{off},{pMHCI}}$ and ${k}_{{off},{pMHCII}}$ the dissociation rate constant of the peptide from MHC I and II molecules, respectively ;$\,{\sigma }_{{NT}}$, ${\sigma }_{{TEM}}$ and ${\sigma }_{{CM}}$ first-order turnover rate constants of naïve, TEM and CM T-cells, ${k}_{{act},{CDi}}$, ${k}_{p,{CDi}}$ and ${k}_{{diff},{CDi}}$ maximum activation, proliferation and differentiation first-order rate constants of T-cells, ${{frc}}_{{TEM}}$ fraction of TEM T-cells differentiating into CM T-cells, ${k}_{G0}$ first-order growth rate constant, ${K}_{G1}$ zero-order growth rate constant, ${{SHR}}_{{Tcell},{TC}1}$ and ${{SHR}}_{{Tcell},{MC}38}$ the vaccine-induced shrinkage effect in TC-1 and MC38 tumor models, respectively; ${{\rm{SHR}}}_{{\rm{trans}},{\rm{BiAb}}}$ and ${{SHR}}_{{per},{BiAb}}$, the transient and persistent BiAb-induced shrinkage effect, respectively, in MC38 tumor model only; ${k}_{d,{nat}}$ first-order natural death rate constant of MC38 tumor, ${k}_{e0,{Tcell}}$ and ${k}_{e0,{Ag}}$ first-order delay rate constants of T-cell and peptide, respectively; ${K}_{{in},{BiAb}}$ zero-order input rate constant. Created in https://BioRender.com.

A multiscale semi-mechanistic PKPD modeling framework was built through the sequential development of sub-models, capturing the key PK and PD processes, along with critical biological interactions required for a vaccine-induced T-cell response and tumor regression, and the temporal interplay between product components (Fig. 2). A data-driven approach was applied, with the model informed by in silico, in vitro, and in vivo data while remaining grounded in knowledge from the literature. Parameter estimates for the framework’s sub-models are provided in Table 1 and Suppl. Tables S1–S4.

**Table 1:** Parameter estimates and their uncertainty (relative standard error, RSE) for the tumor growth dynamic model

Parameters	Description	units	value	RSE %	IAV^a^(%)	RSE%
TC-1 tumor mouse model						
${k}_{G0,{TC}1}$^*e*^	first-order growth rate constant	day^−^^1^	0.605	3.5	22^c^	10
${K}_{G1,{TC}1}$^*e*^	zero-order growth rate constant	mm^3 .^ day^−1^	47.8	12	68^c^	13
${{TS}}_{0,{TC}1}$	tumor volume at the time of cell inoculation	mm^3^	0.05	FIX^d^	—	—
${k}_{d,{TC}1}$	maximum T-cell-induced tumor shrinkage rate constant	day^−1^	0.0837	70	243^c^	17
${{TEM}}_{50,{TC}1}$	T-cells at which half-maximum shrinkage is achieved	cells	1093	2.7	—	—
${{\rm{k}}}_{e0,{Tcell},{TC}1}$	first-order delay rate constant of T-cells effect	day^−1^	0.300	4.1	—	—
${coeff},{TC}1$	exponent of tumor size effect relative to baseline	—	0.183	40	—	—
${RU}{V}_{{TC}1,\exp 1\& 2}$^*b*^	residual unexplained variability in tumor volume in studies 1 and 2	%	36.0	6.9	—	—
${RU}{V}_{{TC}1,\exp 3}$^*b*^	residual unexplained variability in tumor volume in study 3	%	52	5.9	—	—
MC38 tumor mouse model						
${k}_{G0,{MC}38}$	first-order growth rate constant	day^−1^	0.0381	6.2	10	14
$T{S}_{{MC}38,{ss}}$	maximum carrying capacity	mm^3^	47.5 × 10^6^	1.3	—	—
${{TS}}_{0,{MC}38}$	tumor volume at the time of cell inoculation	mm^3^	0.3	FIX^d^	—	—
${k}_{d,{nat},{MC}38,{male}}$	natural death rate constant in males	day^−1^	0.271	11	—	—
${k}_{d,{nat},{MC}38,{female}}$	natural death rate constant in females	day^−1^	0.332	21	27	21
${{\rm{\lambda }}}_{\sup ,{MC}38,{female}}$	exponential decay in the natural tumor death effect in females	day^−1^	0.00465	70	128	86
${k}_{d,{MC}38}$	slope of the T-cell-induced tumor shrinkage rate	day^−1^.cells^−1^	0.0119 × 10^−6^	67	483	34
${{\rm{k}}}_{e0,{Tcell},{MC}38}$	first-order delay rate constant of T-cells effect	day^−1^	0.630	3.5	—	—
${k}_{d,{trans},{BiAb}}$	slope of the transient BiAb-induced tumor shrinkage rate	day^−1^.nM^−1^	1.59 × 10^−3^	4.3	—	—
${K}_{{in},{per},{BiAb}}$	maximum T-cell production at the tumor site	cells.hr^−1^	1	FIX	—	—
${{BiAb}}_{{per},50}$	total BiAb plasma concentration at which half-maximal production is achieved	nM	260	3.9	—	—
${k}_{d,{per},{BiAb}}$	maximum persistent BiAb-induced tumor shrinkage rate constant	day^−1^	0.363	45	370	20
${{TEM}}_{50,{tum}}$	in-situ produced T-cells required to achieve half-maximal shrinkage rate	cells	17.0	4.2	—	—
${{TEM}}_{50,{tum},{trun}}$	in-situ produced T-cells required to achieve half-maximal shrinkage rate with BiAb/truncated pTag-peptide	cells	1000	FIX	—	—
${{\rm{k}}}_{e0,{Ag},{MC}38}$	first-order delay rate constant of free peptide effect	day^−1^	0.286	3.5	—	—
${{slp}}_{{Agfree},{MC}38}$	slope of the peptide stimulatory effect	nM^−1^	15.4 × 10^3^	27	76	141
${coeff},{MC}38$	exponent of tumor size effect relative to baseline	—	−0.189	7.2	—	—
${coeff},{MC}38,{veh}$	exponent of tumor size effect relative to the vehicle	—	0.0585	33	—	—
${RU}{V}_{{MC}38}$^*b*^	residual unexplained variability in tumor volume	%	37	10	—	—

^a^IAV: inter-animal variability ($\sqrt{{\omega }^{2}}$). ^b^Additive residual error model on log-transformed data.^c^−34% correlation between ${k}_{G0,{TC}1}$ and ${K}_{G1,{TC}1}$, −86% correlation between ${k}_{D,{TC}1}$ and ${k}_{G0,{TC}1}$ and 47% correlation between ${k}_{D,{TC}1}$ and ${K}_{G1,{TC}1}$. ^d^the value of tumor volume at the time of cell inoculation was based on the assumption that a volume of 1000 mm^3^ contains 1 × 10^9^ cells^53^. ^e^ ${{TS}}_{{thr}}$ represents the threshold tumor volume at which the tumor growth switches from exponential to linear growth and is expressed as the ratio between ${k}_{G1,{TC}1}$ and ${K}_{G0,{TC}1}$^54^.

### Pharmacokinetic model

Given the mechanism of action of the BiTag drug (Fig. 1A) via delivering its peptide cargo to induce local peptide-specific T-cell activation in the draining lymph nodes following s.c. administration (Fig. 1B), a PK model successfully characterized the lymphatic and systemic disposition of the BiAb and pTag-peptide. This model included five anatomical locations: (1) site of s.c. injection in the hock, (2) hock injection draining popliteal lymph node, (3) hock injection draining inguinal lymph node, (4) site of s.c. injection in the flank, and (5) plasma (i.e., central compartment) (Fig. 2A and S1). The binding kinetics between the BiAb and pTag-peptide were applied in all five locations.

The injection site was initialized with fully saturated BiAb with pTag-peptides (i.e., 1:2) and excess free pTag-peptide in 1:3 preparations. Following the s.c. injection in the hock, an estimated 57% of the absorbed dose entered the popliteal lymph node, while the remaining drained to the inguinal lymph node. The transition kinetics were assumed to be the same for free BiAb and its pTag-peptide conjugate, with an estimated mean transit time of 6.1 h to both lymph nodes. Thereafter, the different forms drained through the efferent lymphatics to rapidly enter the systemic circulation. Upon s.c. administration into the flank, the free BiAb and its pTag-peptide conjugate were absorbed into the central compartment via a nonlinear process. Additionally, free BiAb underwent a parallel first-order absorption process, with a mean absorption time of 34 h. The free pTag-peptide had a fast mean absorption time (0.25 h) from both injection sites. The estimated half-life of conjugate degradation at the injection site was 31 h.

**Fig. 3:**
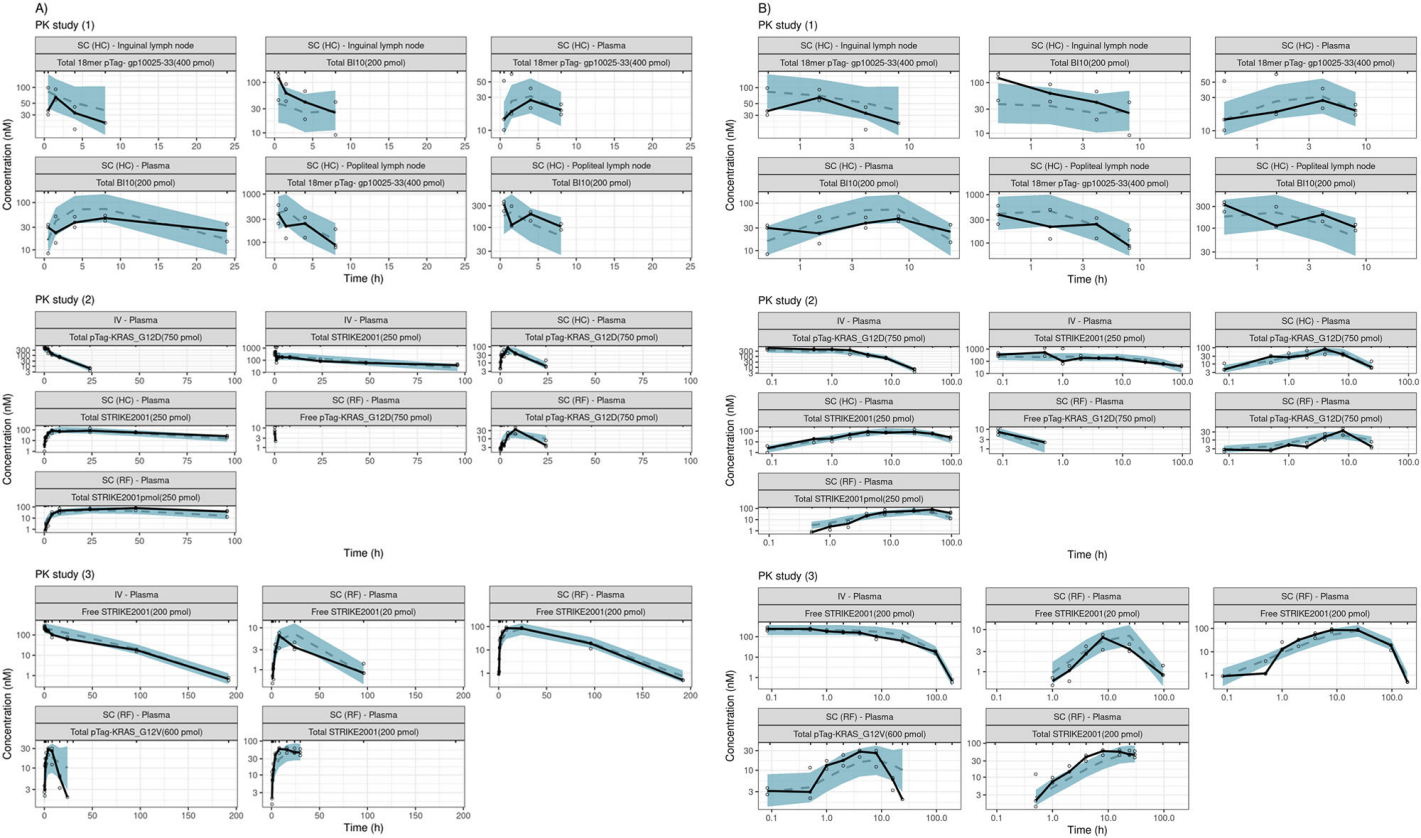
Simulation-based evaluations of the pharmacokinetics model. Simulation-based evaluations of the pharmacokinetics model using 1000 simulations, (**A**) displays time on a linear scale, while (**B**) shows time on a logarithmic scale. Dots illustrate the observed experimental concentrations. The solid and dashed black lines are the median of the observed and simulated concentration-time profiles, respectively. The blue-shaded regions illustrate the 90% confidence interval for the predicted medians based on the simulated data. SC subcutaneous, HC hock, IV intravenous, RF right flank.

Total STRIKE2001 BiAb exhibited significant non-linear elimination from the central compartment, along with linear elimination with an estimated half-life of 29 h. Free pTag-peptide had a short half-life of 23 seconds on average. Clearance and volume of distribution were allometrically scaled by body weight using fixed exponents of 0.75 and 1, respectively, relative to a 20 g reference weight. Across all three PK experiments included in the model analysis, the observed median concentrations were included within the 90% confidence interval around the predicted median (Fig. 3), supporting adequate predictive performance of this sub-model. See Supplementary Material, section 1, for further details.

### Model for the peptide uptake by antigen-presenting cells

Upon reaching the hock injection draining lymph nodes, the BiTag drug binds to CD40 on APCs (e.g., dendritic cells (DCs)) and is internalized via receptor-mediated uptake for peptide delivery (Fig. 1B). The peptide uptake model was applied to describe the two states required for APC-mediated peptide presentation (Eq. 1–4) after the exposure to either the BiAb:pTag-peptide conjugate or its separate components; 1) extracellular peptides, either free or conjugated, attached to APCs, and 2) intracellular released peptides within APCs, where peptide-major histocompatibility complex (MHC) complexes ($p{{MHC}}_{x,y}$, with $x$ indicating class I or II and $y$ denoting popliteal or inguinal lymph nodes) are formed (Fig. 2B). The transition kinetics between the states were informed by a multistate model (Fig. S2), which was developed to characterize the dynamic states of DCs; further details are in Supplementary Material, section 2. There was a close agreement between the in vitro data and model simulations, with the observed median falling inside the 90% confidence interval of the predicted median (Suppl. Fig. S3).

The extracellular attachment of free peptides (${{Ag}}_{{free},y}$) and pTag-peptide conjugated with BiAb (${{Ag}}_{{conj},y}$) to APCs was described by the first-order rate constant (${k}_{{up}}$). Free peptides were internalized directly via a first-order rate constant (${k}_{{tr},{Ag}}$), while conjugated peptides underwent a 6-hour mean delay time through six transit compartments (${{MTT}}_{{tr},{BiAb}:{Ag}}=\frac{6}{{k}_{{tr},{BiAb}:{Ag}}}$), describing a delay, before being released from the conjugate (${{Ag}}_{{conj},{tr},y,n}$) and presented on MHC molecules (${{Ag}}_{{MHC},y}$). The presented peptide decay was influenced not only by APCs’ death rate (${k}_{{APC}}$) but also by the dissociation rate constant of the peptide (${k}_{{off},{pMHCx}}$) from MHC molecules. This dissociation rate depended on epitope sequence and MHC molecule type (Table 2).

1 $$\frac{d({{Ag}}_{{free},{ex},y})}{{dt}}={k}_{{up}}\cdot A{g}_{{free},y}-({k}_{{tr},{Ag}}+{k}_{{APC}})\cdot {{Ag}}_{{free},{ex},y}$$

2 $$\frac{d\left({{Ag}}_{{conj},{ex},y}\right)}{{dt}}={k}_{{up}}\cdot {{Ag}}_{{conj},y}-({k}_{{tr},{BiAb}:{Ag}}+{k}_{{APC}})\cdot {{Ag}}_{{conj},{ex},y}$$

3 $$\frac{d\left({{Ag}}_{{conj},{tr},y,n}\right)}{{dt}}={k}_{{tr},{BiAb}:{Ag}}\cdot {{Ag}}_{{conj},{ex},y}-({k}_{{tr},{BiAb}:{Ag}}+{k}_{{APC}})\cdot {{Ag}}_{{conj},{tr},y,n}$$

4 $$\frac{d({{Ag}}_{{MHC},y})}{{dt}}={k}_{{tr},{Ag}}\cdot {{Ag}}_{{free},{ex},y}+{k}_{{tr},{BiAb}:{Ag}}\cdot {{Ag}}_{{conj},{tr},y,{NN}}-({k}_{{off},{pMHCx}}+{k}_{{APC}})\cdot {{Ag}}_{{MHC},y}$$

**Table 2:** Summary of binding affinities

Parameter	18mer pTag- gp100_25-33_^a^	pTag-KRAS_G12D^b^	pTag-KRAS_G12V^b^	pTag-HPV-16 E7_44-62_^b^	pTag- gp100_25-33_^b^	pTag-OVA_323-339_^b^	pTag-ADPGK^b^	pTag-KRAS_G12V^b^
${k}_{{on}}$ (nM^-1^.hr^-1^)^c^	2.3	0.576	0.576	0.576	0.576	0.576	0.576	0.576
${k}_{{off}}$ (hr^-1^)^c^	2.74	0.259	0.259	0.259	0.259	0.259	0.259	0.259
${K}_{D,{MHCI}}$(nM)^d^	—	—	—	59.9	154.9	—	3.92	2795
${K}_{D,{MHCII}}$(nM)^d^	—	—	—	—	—	401.8	—	—

^a^peptide tagged to BI10. ^b^peptide tagged to STRIKE2001. ^c^The association constants (${k}_{{on}}$) and dissociation rate constants (${k}_{{off}}$) between the bispecific antibody and p-tag peptide were fixed to predetermined values using surface plasmon resonance (SPR)^17,18^. ^d^ Potential peptide epitopes and MHC-I or MHC-II affinities for mice were obtained based on netMHCpan 4.0^55^.

### T-cell dynamics model

Depending on the peptide, it is presented on MHC-I or MHC-II to activate CD8^+^ or CD4^+^ T-cells, respectively (Fig. 2B). The T-cell dynamics model evaluated the T-cell expansion, following the BiAb:pTag-peptide conjugate or its separate entities administration, in the hock injection draining lymph nodes (Figs. 1B, 2B). Naïve T-cell (${{NT}}_{y}$) followed a homeostasis assumption (Eq. 5), with turnover rate (${\rho }_{{NT}})$. Its activation rate (${{\rm{\sigma }}}_{{NT},{CDi}}$, with $i$ indicating CD8^+^ or CD4^+^ T-cells) to transition effector memory T-cells (${{TEM}}_{{CDi},y}$) was triggered by interacting with $p{{MHC}}_{x,y}$, calculated as ${{Ag}}_{{MHC},y}$ multiplied by ${Avogadr}{o}^{{\prime} }{snumber}$.

5 $$\frac{d\left({{NT}}_{y}\right)}{{dt}}={\rho }_{{NT}}\cdot \left({{NT}}_{y,0}-{{NT}}_{y}\right)-{{\rm{\sigma }}}_{{NT},{CDi}}\cdot f\left(p{{MHC}}_{{xy}}\right)\cdot {{NT}}_{y}$$

${{TEM}}_{{CDi},y}$ were eliminated at a first-order death rate constant ${\rho }_{{TEM},{CDi}}$. Upon interacting with $p{{MHC}}_{x,y}$, they expanded with the proliferation rate constant ${k}_{p,{CDi}}$ and converted into central memory T-cells (*CM*_*CDi,y*_) via the differentiation rate constant ${k}_{{diff},{CDi}}$. Their differentiation was related to the peptide-MHC complex saturation function $f\left(p{{MHC}}_{x,y}\right)$ with σ_50_ representing the number of ${\rm{p}}{{\rm{MHC}}}_{{\rm{x}},{\rm{y}}}$ required for half-maximal effect. When $f\left(p{{MHC}}_{x,y}\right)$ approached 0, a fraction of effector cells (${fr}{c}_{{TEM}}$=0.1) differentiated into ${{CM}}_{{CDi},y}$ (Eq. 6).

6 $$\frac{d\left({{TEM}}_{{CDi},y}\right)}{{dt}}=\left({\sigma }_{{NT},{CDi}}\cdot {{NT}}_{y}+{k}_{p,{CDi}}\cdot {{TEM}}_{{CDi},y}+{k}_{{diff},{CDi}}\cdot {{CM}}_{{CDi},y}\right)\cdot f\left(p{{MHC}}_{{xy}}\right)-\left({k}_{{diff},{CDi}}\cdot \left(1-f\left(p{{MHC}}_{x,y}\right)\right)\cdot {fr}{c}_{{TEM}}+{\rho }_{{TEM},{CDi}}\right)\cdot {{TEM}}_{{CDi},y}$$

**Fig. 4:**
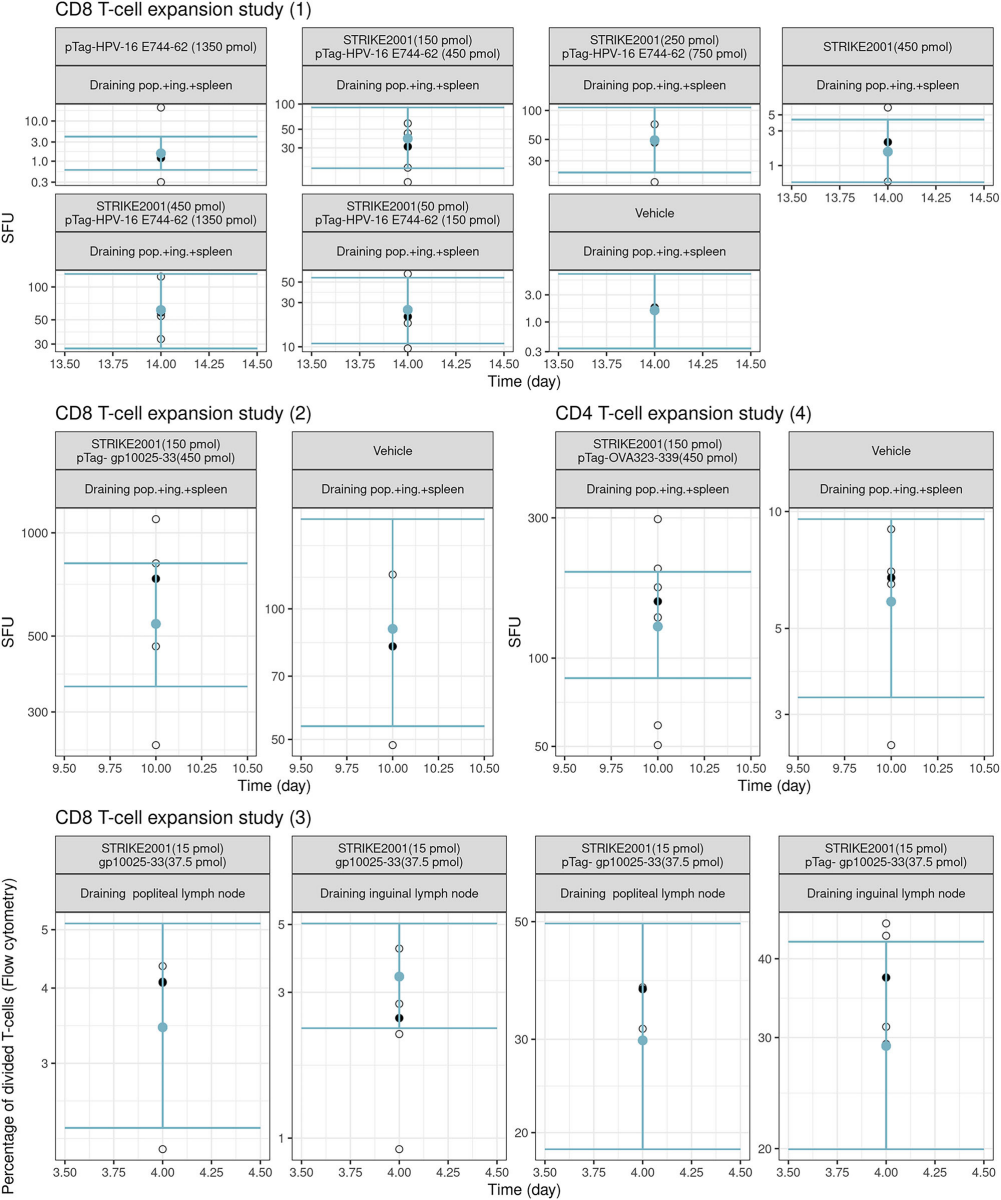
Simulation-based evaluations of the T-cell dynamics model. Simulation-based evaluations of the T-cell dynamics model using 1000 simulations. The black open dots are the observed experimental T-cell measurements across the sample collection sites. The black solid dots are the median of the observed data. The blue solid dots are the medians of the simulated data. The blue-standard errors are the 90% confidence interval of the predicted medians based on the simulated data.

Similarly, ${{CM}}_{{CDi},y}$, with a death rate constant ${\rho }_{{CM},{CDi}}$, proliferate and differentiate into ${{TEM}}_{{CDi},y}$ (Eq. 7). ${\sigma }_{{NT},{CDi}}$, ${k}_{p,{CDi}}$, and ${k}_{{diff},{CDi}}$ were assumed equal, estimated at 0.63 and 0.98 day^−^^1^ for CD8^+^ and CD4^+^ T-cells, respectively. The simulation-based 90% confidence interval for the predicted median encompasses the observed median of the four in-vivo T-cell expansion involved in the analysis, indicating satisfactory model predictive ability (Fig. 4). See Supplementary Material, section 3, for details.

7 $$\frac{d\left({{CM}}_{{CDi},y}\right)}{{dt}}={k}_{{diff},{CDi}}\cdot \left(1-f\left(p{{MHC}}_{x,y}\right)\right)\cdot {fr}{c}_{{TEM}}\cdot {{TEM}}_{{CDi},y}+{k}_{p,{CDi}}\cdot f\left(p{{MHC}}_{x,y}\right)\cdot {{CM}}_{{CDi},y}-\left({k}_{{diff},{CDi}}\cdot f\left(p{{MHC}}_{x,y}\right)+{\rho }_{{CM},{CDi}}\right)\cdot {{CM}}_{{CDi},y}$$

### Tumor growth dynamics model

Natural tumor growth was best captured by exponential-linear and Gompertz models for the TC-1 and MC38 tumor models, respectively (Fig. 2B). In the TC-1 model, ${{\rm{k}}}_{G0,{TC}1}$ and ${{\rm{K}}}_{G1,{TC}1}$ represented the first-order and zero-order growth rate constants, with ${{\rm{TS}}}_{{thr}}$ governing the tumor volume threshold for exponential-to-linear growth switch. The MC38 model included the first-order growth rate constant (${{\rm{k}}}_{G,{MC}38}$), with a maximum carrying capacity ($T{S}_{{MC}38,{ss}}$), and a natural death rate (${k}_{d,{nat},{MC}38}$), with females exhibiting a 1.2-fold higher death rate than males. However, the natural death in females decayed exponentially over time (${{\rm{\lambda }}}_{\sup ,{MC}38}$) with a half-life of 149 days and inter-animal variability (IAV) of 127%.

To achieve the tumor regression, ${{TEM}}_{{CD}8}$ enter the circulation and infiltrate into the tumor microenvironment (Fig. 2B). The vaccine peptide-induced tumor shrinkage was mediated by ${{TEM}}_{{CD}8}$ dynamics, where an effect delay (${{TEM}}_{{del},{CD}8,z}$, where $z$ represents a specific mouse model) was incorporated to best capture the data. This delay reflected both the latency before ${{TEM}}_{{CD}8}$ exerting their antitumor effect and the persistence of this effect, with estimated delay half-lives $(\frac{\mathrm{ln}(2)}{{{\rm{k}}}_{e0,{Tcell},z}})$ of 2.3 and 1.1 days for TC-1 and MC38, respectively (Eq. 8). In the TC-1 model, ${{TEM}}_{{del},{CD}8}$ decay rate increased with larger tumor size, whereas in MC38, it decreased. Additionally, in MC38, free peptides in plasma ($A{g}_{{free},p,z}$) increased ${{TEM}}_{{del},{CD}8}$, following an estimated effect delay half-life of 2.4 days (Eqs. 8 – 9).

8 $$\frac{d\left({{TEM}}_{{del},{CD}8,z}\right)}{{dt}}={{\rm{k}}}_{e0,{Tcell},z}\cdot \left({{TEM}}_{{CD}8,z}\cdot \left(1+{{slp}}_{{Agfree},z}\cdot A{g}_{{del},{free},p,z}\right)-{{TEM}}_{{del},{CD}8,z}\cdot {\left(\frac{T{S}_{z}}{{\mathrm{TS}}_{0,z}}\right)}^{{{coeff}}_{z}}\right)$$

9 $$\frac{d\left(A{g}_{{del},{free},p,z}\right)}{{dt}}={{\rm{k}}}_{e0,{Ag},z}\cdot \left(A{g}_{{free},p,z}-A{g}_{{del},{free},p,z}\right)$$

Where ${{TS}}_{0,z}$ and ${{TS}}_{z}$ are tumor sizes at time=0 and time=t, respectively, ${{\rm{coeff}}}_{z}$ the tumor size effect exponent, and ${{slp}}_{{Agfree},z}$ is the slope of the stimulatory effect of $A{g}_{{del},{free},p,z}$.

For the TC-1 model, the maximum shrinkage rate constant ${k}_{d,{TC}1}$ was 0.084 day^−^^1^, with a half-maximal effect estimated at an ${{TEM}}_{50,{TC}1}$ of 1093 cells (Eq. 10). In the MC38 model, ${k}_{d,{MC}38}$ defined the slope of the shrinkage rate as a function of ${{TEM}}_{{del},{CD}8,{MC}38}$ (Eq. 11) (Figs. 1B and 2B).

In the MC38 model, two distinct BiAb-induced tumor shrinkage effects were identified: transient and persistent. For the transient effect, ${k}_{d,{trans},{BiAb}}$ represents the slope of the shrinkage rate as a function of the total BiAb plasma concentration (${BiA}{b}_{{total},p}$) (Eq. 11). The persistent effect, observed only after high BiAb dose administration (i.e., 450 pmol), was described by Eqs. 11 – 12, where ${K}_{{in},{per},{BiAb}}$ denotes the maximum T-cell production at tumor site, ${{BiAb}}_{{per},50}$ denotes the concentration at which half-maximal production is achieved, ${k}_{d,{per},{BiAb}}$ represents the maximum shrinkage rate and ${{TEM}}_{50,{tum}}$ represents the in-situ produced T-cell number required to achieve half-maximal rate. ${{TEM}}_{50,{tum}}$ was >50-fold higher for the BiAb/truncated p-Tag-peptide mixture, compared to BiAb alone or in a conjugate. Additionally, the tumor growth rate was lower when the MC38 tumor volume decreased relative to the vehicle control. For STRIKE2001:pTag-KRAS_G12V mixture, no vaccine peptide specific T-cell expansion was assumed.

10 $$\frac{d\left(T{S}_{{TC}1}\right)}{{dt}}={{\rm{k}}}_{G0,{TC}1}\cdot T{S}_{{TC}1}\cdot \left(1-\frac{{{TS}}_{z}^{20}}{{{{TS}}_{{thr}}^{20}+{TS}}_{z}^{20}}\right)+{{\rm{K}}}_{G1,{TC}1}\cdot \left(\frac{{{TS}}_{z}^{20}}{{{{TS}}_{{thr}}^{20}+{TS}}_{z}^{20}}\right)-\frac{{k}_{d,{TC}1}\cdot {{TEM}}_{{del},{CD}8,{TC}1}}{{{TEM}}_{50,{TC}1}+{{TEM}}_{{del},{CD}8,{TC}1}}\cdot T{S}_{{TC}1}$$

11 $$\frac{d\left(T{S}_{{MC}38}\right)}{{dt}}={k}_{G0,{MC}38}\cdot T{S}_{{MC}38}\cdot \mathrm{ln}\left(\frac{T{S}_{{MC}38,{ss}}}{T{S}_{{MC}38}}\right)\cdot {\left(\frac{T{S}_{{MC}38}}{T{S}_{{MC}38,{veh}}}\right)}^{{coeff},{MC}38,{veh}}-\left({k}_{d,{nat},{MC}38}\cdot {e}^{\left(-{\lambda }_{\sup ,{MC}38}* {time}\right)}+{k}_{d,{MC}38}\cdot {{TEM}}_{{del},{CD}8,{MC}38}+{k}_{d,{trans},{BiAb}}\cdot {BiA}{b}_{{total},p}+\frac{{k}_{d,{per},{BiAb}}\cdot {Tcel}{l}_{{tum}}}{{{TEM}}_{50,{tum},{mix}}+{Tcel}{l}_{{tum}}}\right)\cdot T{S}_{{MC}38}$$

12 $$\frac{d\left({Tcel}{l}_{{tum}}\right)}{{dt}}=\frac{{K}_{{in},{per},{BiAb}}\cdot {BiA}{b}_{{total},p}}{{{BiAb}}_{{per},50}+{BiA}{b}_{{total},p}}$$

**Fig. 5:**
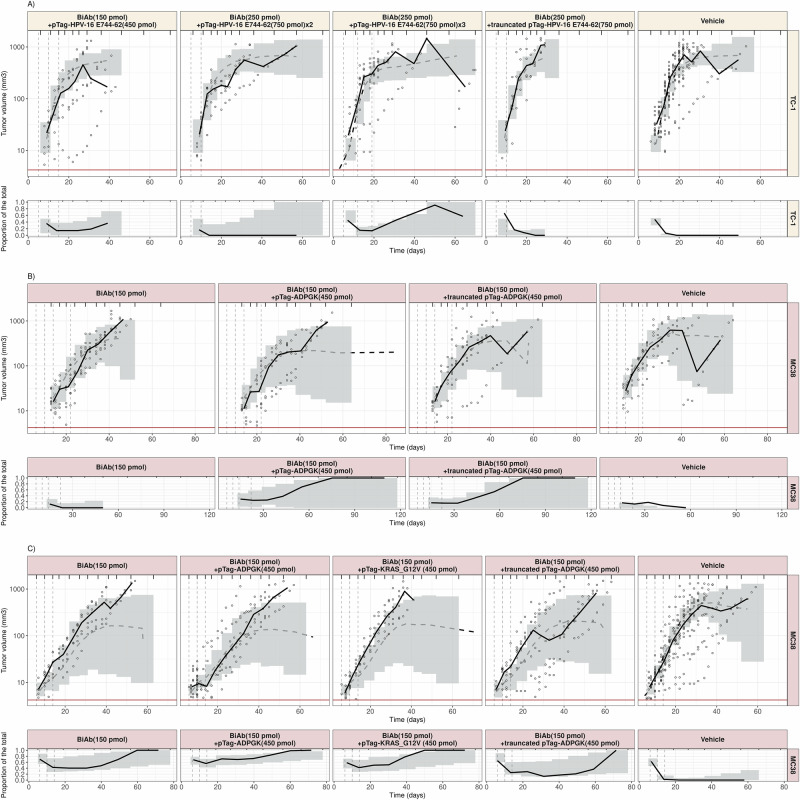
Simulation-based evaluations of the tumor growth dynamics model. Simulation-based evaluations of the tumor growth dynamics model for the TC-1 tumor model (**A**), the MC38 tumor model with fixed repeated dosing (**B**), and a second high-dose regimen (**C**). Animals with tumors > 1000 mm^3^ were sacrificed and thereafter treated as censored in the analysis. The black dots are the observed experimental tumor volumes. The solid and dashed black lines are the median of the observed and simulated tumor volumes, respectively, for the data above the limit of quantification only (4.2 mm^3^). Gray-shaded areas are the 90% confidence intervals of the predicted medians constructed based on 1000 datasets simulated from the model, for the data above the limit of quantification only. Lower panels show the observed proportions below the lower limit of quantification (solid lines), where shaded areas depict the 90% confidence intervals of these proportions based on the simulated tumor volumes.

The simulation-based evaluations shown in Fig. 5 demonstrate that the 90% confidence intervals of the predicted medians cover the observed median of tumor volume data, indicating good predictive model performance.

### Model applications

The developed modeling framework enabled exploration of key design questions relevant to optimizing SLP-based vaccines through informed formulation strategies that support both efficient drug delivery and robust immune activation. Here, we illustrated how model-based simulations were used to generate insights on the impact of conjugate stability on tumor response. Increasing binding affinity (i.e., lower ${k}_{{off}}$) between pTag and anti-pTag improved in vivo conjugate stability, leading to prolonged total peptide plasma residence time (Fig. S4) and enhanced T-cell expansion in lymph nodes (Fig. S5). The impact of varying the ${k}_{{off}}$ on the median of tumor volume-time profiles is shown in Figs. S6, S7, demonstrating that higher binding affinity enhances tumor regression, with the extent of improvement depending on the tumor model, dose level, and dosing frequency.

**Fig. 6:**
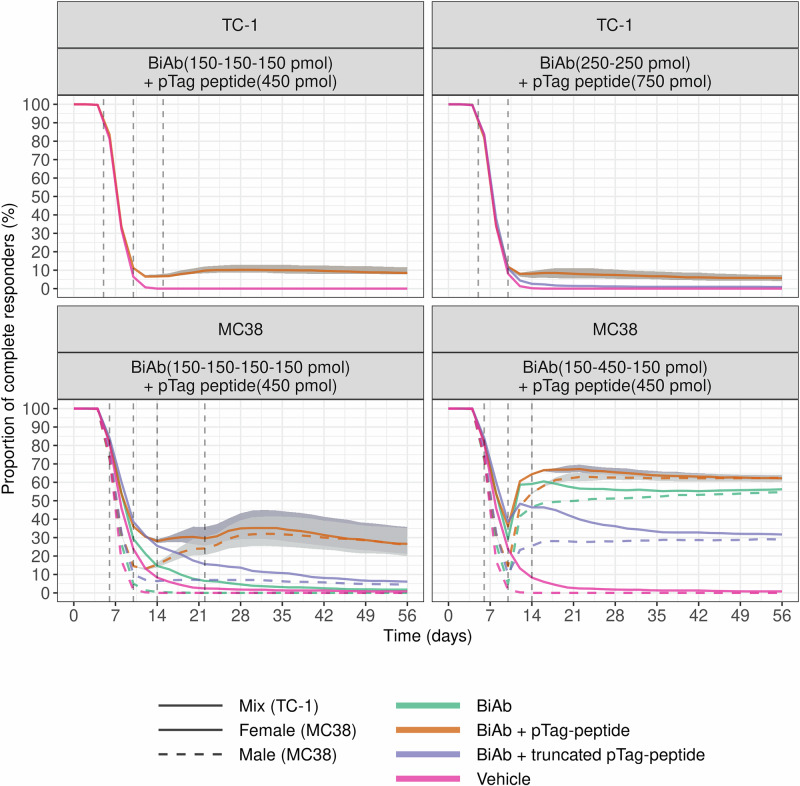
Model-simulated proportions of complete responders in tumor models. The model simulated proportions of complete responders, defined as the proportion of mice with undetectable tumor volumes, in TC-1 and MC38 tumor models over time, following treatment with BiAb (STRIKE2001) and pTag-peptide (pTag-HPV-16 E744-62 in TC-1 and pTag-ADPGK in MC38). The vertical dashed lines indicate the dosing times. The shaded areas were constructed based on varying the binding affinity by 0.25- and 4-fold. In the period before treatment initiation, 100% complete responders were recorded, as tumor cells were inoculated at time 0 and were therefore not yet measurable.

On day 30, the impact of binding affinity change factors between 0.25 and 4 on response rates was explored under various scenarios. A BiAb prime dose of 150 pmol and pTag-peptide at 450 pmol, followed by two booster doses, yielded a complete response rate ranging between 9 and 13% of the TC-1 tumor-bearing mice and partial tumor reduction (i.e., > 50% relative to controls) in 21–35% of the mice (Fig. 4 and S8). Increasing the prime dose to 250 pmol BiAb and 750 pmol pTag-peptide with a single booster lowered complete response to 5–10% and partial response to 11–19%. The BiAb:pTag-peptide had approximately 10-fold higher complete response rates compared to the BiAb with truncated pTag-peptide. For the MC38 tumor, administration of a BiAb prime dose of 150 pmol with 450 pmol of pTag-peptide, followed by three booster doses, resulted in complete response rates of 29–45% in females and 26–41% in males (Fig. 6). Partial tumor reduction was observed in over 90% of mice (Fig. S8). Increasing the second BiAb dose to 450 pmol (a 1:1 BiAb:pTag-peptide ratio) improved complete response rates to 64–68% and 61–66% in females and males, respectively, with partial responses observed in approximately 99% of mice, despite reducing the regimen to two booster doses. The BiAb:pTag-peptide had over 9-fold and 3-fold higher complete response rates compared to BiAb alone or with truncated pTag-peptide, respectively; the differences narrowed to 1.2-fold and 2-fold by increasing the BiAb second dose to 450 pmol across the different formulations. In the MC38 model, female mice showed a higher proportion of complete responders than males during the first month; however, this difference diminished over time (Fig. 6). Figure S9 shows that IAV was higher in female mice than in male mice. Figure S10 illustrates that the contribution of vaccine peptide-induced tumor shrinkage to the overall antitumor effect was reduced at high BiAb doses, and that the persistent BiAb effect diminished in the presence of the truncated pTag-peptide.

## Discussion

This work was motivated by a central translational question in cancer vaccination: how the magnitude, duration, and spatial distribution of antigen exposure determine the quality of T-cell priming and downstream tumor control. By developing a novel, semi-mechanistic PKPD modeling framework characterizing the complex, dynamic immune responses elicited by transforming SLP vaccines into Antibody Drug Conjugate designs, we were able to use simulations to connect molecular design choices in an antibody-conjugated vaccine to organism-level therapeutic outcomes across distinct tumor immune contexts. While grounded in data from two bispecific CD40-targeting antibodies with similar designs, the framework was developed to be modular and generalizable for other ADC-based therapies with the same indication, providing a structure for integrating diverse data types. The framework comprises four interconnected sub-models: (1) PK model, (2) APC peptide uptake model, (3) T-cell dynamics model, and (4) tumor growth dynamics (TGD) model, quantifying the interplay between the anti-CD40 BiAb, the peptides, APCs, T-cells, and tumor dynamics. The PK model characterized linear and non-linear BiAb and pTag-peptide kinetics across s.c. and intravenous (i.v.) administration. The APC uptake model captured the delayed peptide presentation via MHC molecules when administered in the conjugate form. The T-cell dynamics model quantified T-cell activation, proliferation, and differentiation based on the peptide-MHC interaction. The TGD model characterized the anti-tumor effects of BiAb:pTag-peptide conjugate, BiAb alone, and BiAb/truncated pTag-peptide mixture in non-inflamed and inflamed tumors. Model structures were designed to align with existing biological knowledge while also being informed by available data, ensuring both mechanistic relevance and data-driven adaptability.

The integration of various data types (i.e., binding affinities, PK profiles, peptide uptake by APCs, T-cell expansion, and tumor response) is a key strength that differentiates this work from the existing semi-mechanistic models in immuno-oncology with the cancer vaccine context. While the prior models rely primarily on tumor size data^23–25^, this framework enables more mechanistically anchored and data-informed predictions. By the induced immune response against tumors, the model supports the learn-and-confirm cycle of MIDD, offering a platform for both hypothesis generation and experimental refinement. Furthermore, it enhances understanding of how various neoantigen peptides can be used in vivo, even when they are unstable or exhibit low MHC-binding affinity, without compromising biological activity through conjugation-based delivery.

The framework captured the administration route-dependent differences in lymphatic drainage and systemic exposure. After s.c. injection in the hock, the BiAb:pTag-peptide conjugate drained to popliteal and inguinal lymph nodes in parallel^26^ with a mean transition time of 6 h, and subsequently into systemic circulation. However, for s.c. injection in the flank, an unquantified transition rate was assumed owing to the lack of lymph node concentration data. Our results indicated that the free peptide was rapidly absorbed from both injection sites and exhibited a very short half-life, compared to its conjugated form, which displayed prolonged apparent stability. An estimated 66% of the BiAb dose (i.e., either free or in a conjugate form) was absorbed into the central circulation, consistent with reported human monoclonal antibody bioavailability values following s.c. administration (52–80%)^27^. The identified saturable absorption kinetics into blood after s.c. administration in the flank may reflect solubility limitations or saturated transport processes^28^. Additionally, the conjugate degradation rate at the injection site could be due to tissue trapping or bypassing FcRn binding, thereby contributing to reduced bioavailability^29^.

A key finding is that conjugation of SLPs to the CD40-targeted BiAb fundamentally altered peptide disposition. Free peptide was rapidly absorbed and eliminated, whereas the BiAb:pTag-peptide conjugate exhibited prolonged apparent stability and lymphatic recirculation. This extended residence time translated into delayed but sustained peptide presentation by APCs, consistent with receptor-mediated uptake and intracellular release rather than passive diffusion. These features directly address one of the major limitations of peptide vaccines in the clinic: insufficient and short-lived antigen availability at the sites of T-cell priming.

The PK component of the framework provided a mechanistic insight by considering the lymphatic drainage for therapeutic delivery. Drainage of the conjugate from the s.c. injection site in the hock to the systemic circulation occurred via the lymphatic system with a mean transit time of ~6 h, consistent with the preferential lymphatic transport of large proteins after s.c. injection^30^. An estimated 57% of the absorbed dose reached the popliteal lymph node, with the remainder entering the inguinal lymph node, in line with previous reports showing lymphatic drainage from s.c. injection, in the footpad of the hindleg, is evenly distributed between the two nodes^26^. The estimated bioavailability (66%) and target-mediated clearance were in line with known monoclonal antibody behavior, supporting the biological plausibility of the exposure patterns driving the downstream immune response^31^.

Within our framework, APCs, in the draining lymph nodes, played a central role in initiating the immune response, as APCs expressing CD40, such as dendritic cells, monocytes, macrophages, and B-cells, mediate this response by presenting vaccine peptides on MHC-I molecules to stimulate CD8^+^ T-cells (i.e., via cross-presentation) and on MHC-II molecules to activate CD4^+^ T-cells^18^. The APC uptake model revealed that conjugation created a temporal separation between vaccine administration and peptide-MHC availability, in contrast to the rapid but fleeting exposure from the naked peptide. This delay likely reflects receptor-mediated internalization of the anti-CD40 antibody and gradual intracellular release of the pTag-peptide.

The draining lymph nodes were the sites for BiAb:pTag-peptide uptake by APCs and subsequent T-cell activation, given that large biomolecules ( < 500 kDa) undergo highly efficient fluid-phase transcytosis through the sinus floor to reach the lymph node parenchyma intact^32^. Despite the sparse T-cell expansion data, the inferred activation and proliferation kinetics for CD4^+^ and CD8^+^ T-cells, 0.98 and 0.63 day^−^^1^, respectively, are in a reasonable agreement to the reported activated CD4^+^ T-cell proliferation rate constant ( ≈ 0.60 day^-1^)^33^, and ≈1.5 and ≈2.1 day^−1^ reported for CD4^+^ and CD8^+^ T-cells, respectively, during the initial expansion phase^34^. The different (naïve, TEM, CM) T-cells phenotypes were further differentiated via distinct death-rate constants^35^. Importantly, the model predicts that it is not simply the amount of antigen delivered, but its persistence in the APC–T-cell interface that governs the magnitude and durability of the response.

A major strength of this work is the explicit comparison of non-inflamed (TC-1) and inflamed (MC38) tumor models. These represent biologically distinct immune ecosystems, and the framework revealed that they respond to vaccination through different mechanisms. Since the T-cells target the cancer cells expressing tumor-associated/-specific antigens^1^, the vaccine peptide-induced tumor shrinkage in the model was driven by the vaccine peptide-specific T-cells. In both tumor models used, CD8 + T-cells were considered the primary mediators of tumor shrinkage. The choice of antigens was based on previously published data, for which no confirmed CD4 epitopes have been reported, and by using TCR sequencing, we have not noted an expansion in CD4 T-cell clones in response to the treatment (manuscript in preparation).

In TC-1 tumors, which have low baseline immune infiltration^36^, antigen delivery was critical. The BiAb:pTag-peptide conjugate produced substantially greater tumor control than the BiAb/truncated peptide mixture, despite identical CD40 agonism (Fig. 6). This indicates that CD40 stimulation alone is insufficient in immune-cold tumors; sustained, targeted antigen delivery is required to drive effective priming and tumor killing. Additionally, the absence of adjuvant conjugation for the SLP (i.e., truncated pTag-peptide) and its rapid elimination further contribute to the lack of response, as seen in the TC-1 model and previous studies^23^, while showing some antitumor effect with high variability in the MC38 model (Fig. S7). In the MC38 model, the BiAb alone, at a low dose, induced a transient anti-tumor response, likely mediated by myeloid cell infiltration^37^. At higher doses, an additional persistent treatment effect was noted, possibly due to strong CD40 stimulation near the tumor microenvironment, where antigen shedding occurs, harnessing the tumor itself as a “vaccine”. However, this effect was attenuated with the truncated pTag-peptide (Fig. S9), likely due to T-cell anergy^38^, a phenomenon observed in a phase 3 study on metastatic melanoma. The study found that adding a gp100 peptide vaccine to ipilimumab (a CTLA-4 inhibitor) did not improve its efficacy and instead showed a trend toward reduced response compared to ipilimumab alone^39^. Additionally, sex emerged as a significant covariate, with females exhibiting a 20% higher natural death rate and greater IAV compared to males (Fig. S10), suggesting potential sex-based differences in the tumor microenvironment. Ray et al.^40^ reported that female murine models of metastatic colorectal cancer exhibited increased T-cell infiltration and IL-10⁺ macrophages, features associated with improved survival. The quantified variability in the net natural tumor growth likely stems from the young age of the mice (mostly 8 weeks), which may reflect differences in immune system maturation. These findings illustrate how the same vaccine formulation can have fundamentally different consequences depending on tumor immune-microenvironment.

Beyond descriptive modeling, simulations using this framework highlighted mechanistic relationships of translational interest. The simulations showed that higher binding affinity is linked to improved anti-tumor response and responder rates, with the magnitude of improvement varying by tumor model, dose level, and dosing frequency (Fig. 6). Increasing the affinity also prolonged the apparent residence time of total pTag-peptide in the circulation (Fig. S4), potentially enhancing immune engagement via lymphatic recirculation. This directly supports the hypothesis that antigen exposure duration is a key driver of therapeutic efficacy. Similarly, simulations revealed that in TC-1 tumors, increasing the dose with fewer boosters reduced efficacy, suggesting that limited memory development and insufficient T-cell expansion may hinder tumor shrinkage. The BiAb:pTag-peptide conjugate achieved markedly higher complete response rates than BiAb/truncated pTag-peptide mixture (Fig. 6). This highlights the potential relevance in “cold” tumors (e.g., TC-1), where endogenous antigen is scarce, as ADAC actively targets antigen to APCs to enhance presentation and de novo T-cell priming. However, these differences diminished when the BiAb second dose was increased across formulations in the MC38 model. This may reflect the dual role of adjuvants, which can act solely as an adjuvant or also exert direct antitumor effects, depending on the administered dose. Since SLP vaccines vary widely in composition and are often not evaluated for stability, our simulations demonstrated how targeted drug delivery through Antibody Drug Conjugate designs may offer a strategy to enhance vaccine anti-tumor responses (Fig. 6).

Together, these results demonstrate how molecular design (affinity), formulation (conjugation), and clinical strategy (dose and schedule) jointly determine immune activation and tumor control, revealing interactions that are difficult to disentangle experimentally but become transparent through simulation. By quantitatively linking these design dimensions, the framework supports a precision medicine approach, in which vaccine constructs and dosing regimens can be tailored to tumor immune context and desired exposure profiles. Our findings demonstrate that ADC-based vaccine delivery can transform unstable, weakly immunogenic peptides into effective immunotherapies by reshaping antigen exposure in space and time. The multiscale framework provides a quantitative bridge between peptide chemistry, antibody engineering, lymphoid biology, and tumor control, aligning with emerging digital-twin concepts in precision medicine and translational oncology^21,22^. Although developed for the ADAC platform, the model captures key processes that may also be relevant to other subunit cancer vaccines. It describes how pharmacokinetic changes introduced by drug-conjugate design, compared with SLP (antigen) and adjuvant mixtures, influence immunological pharmacodynamic responses through effects on antigen availability, APC uptake, processing, and presentation, and ultimately the magnitude and duration of antigen-specific T-cell responses. Extension to other modalities (e.g., mRNA neoantigen vaccines) would primarily require adapting formulation-dependent antigen input/release and accounting for adjuvant effects through changes in antigen presentation efficiency and/or the magnitude and time-course of immune activation. These types of extrapolations may require modality-specific parameter re-estimation and validation with relevant data. The high variability in effect-related parameters may stem from factors that are not fully captured by the sub-models, such as prior IAV in exposure, baseline peptide-specific naïve T-cell levels, the development of anti-drug antibodies (i.e., human IgG in murine host), immunosuppressive mechanisms, and T-cell expansion and killing capacity. Despite the small number of animals in a few studies, the nonlinear mixed-effects approach strengthens the analysis by allowing sparse data to be integrated across studies/sources and by separating typical behavior from unexplained variability. This approach also enables analysis with fewer data points per individual, whereas a larger sample size may have been required with traditional statistical methods. Although there was no lymph node PK sampling for STRIKE2001, lymph node distribution was informed using BI10 lymph node PK data, given their similar design and construct characteristics. A temporal separation between peptide arrival and peptide–MHC availability is empirically represented using a series of transit compartments informed by in vitro data rather than in vivo measurements. Furthermore, the peptide entry into the release compartment, within APC, was assumed to directly drive naïve T-cell activation, without an explicit delay for MHC loading and presentation, as available data did not support identification of an additional presentation-delay process. The lack of exhaustion phenotyping/functional profiling data prevented the assessment of therapy-induced T-cell dysfunction under prolonged antigen exposure. Additionally, the model did not capture the persistence of APC–T-cell interactions^41^ or the involvement of multiple CD40-expressing APC populations, due to the lack of sufficient experimental data. While these limitations highlight areas for future model refinement, they also underscore the value of a flexible, mechanistically structured framework that can be readily extended as new data emerge, enabling broader applicability to future ADAC technology platforms.

This study presents a multiscale semi-mechanistic PKPD modeling framework integrating in silico, in vitro, and in vivo data to characterize immune-tumor interactions and vaccine effects. The framework has the capacity to generate biologically meaningful predictions, particularly by capturing the dynamic interplay among components of the ADAC platform. The model-based simulations highlight the importance of conjugating the SLP vaccine to the developed BiAb (STRIKE2001) to achieve the desired therapeutic effects. Additionally, increasing the pTag binding affinity prolonged plasma peptide residence and enhanced tumor regression to various degrees in TC-1 and MC38 tumor models. Specifically, the benefit of pTag–peptide in MC38 was BiAb dose- and context-dependent. This framework offers a valuable MIDD tool for precision design of cancer vaccines through optimizing dosing schedules, binding affinities, BiAb:pTag-peptide ratios, and exchange of the drug cargo in future studies.

## Methods

### Experimental data and studies design

Several studies have been conducted to support the preclinical evaluation and were used to inform the development of our framework. For further details, we refer the reader to previous publications^17,18^. An overview of the vaccine mixtures and dosing schedules is provided in Table 3. These studies included PK, in vitro peptide uptake and release assay, T-cell expansion, and tumor growth experiments.

**Table 3:** Experimental summary table

Study number	Mouse strain	Number of observations^d^	Treatment groups (number of animals per group)	Bispecific antibody	pTag-peptide	Immunization day
In vivo pharmacokinetic studies						
1	C57BL/6	70	1 (*n* = 3)	Bi10^c^ (200pmol)	18mer pTag- gp100_25-33_ (400pmol)	0
2	tghCD40	141	1 (*n* = 3)	STRIKE2001 (250 pmol)	pTag-KRAS_G12D (750pmol)	0
			2 (*n* = 3)	—	pTag-KRAS_G12D (750pmol)	
3	tghCD40	112	1 (*n* = 3)	STRIKE2001 (200pmol)	pTag-KRAS_G12V (600pmol)	0
			2 (*n* = 3)	STRIKE2001 (200pmol)	—	
			3 (*n* = 3)	STRIKE2001 (20pmol)	—	
In vitro peptide uptake and release assay						
1	-	192	1	Bi17^c^ (300 nM)	fTag-CMV pp65_495-503_ (900 nM)	0
			2	Bi17^c^ (300 nM)	—	
			3	-	fTag-CMV pp65_495-503_ (900 nM)	
In vivo T-cell expansion studies						
1	tghCD40	23	1 (*n* = 4)	STRIKE2001 (50pmol)	pTag-HPV-16 E7_44-62_ (150pmol)	0, 5,10
			2 (*n* = 4)	STRIKE2001 (150pmol)	pTag-HPV-16 E7_44-62_ (450pmol)	
			3 (*n* = 4)	STRIKE2001 (250pmol)	pTag-HPV-16 E7_44-62_ (750pmol)	
			4 (*n* = 4)	STRIKE2001 (450pmol)	pTag-HPV-16 E7_44-62_ (1350pmol)	
			5 (*n* = 4)	STRIKE2001 (450pmol)	-	
			6 (*n* = 4)	-	pTag-HPV-16 E7_44-62_ (1350pmol)	
2	tghCD40	8	1 (*n* = 3)	STRIKE2001 (150pmol)	pTag- gp100_25-33_ (450pmol)	0, 5
3	tghCD40	15	1 (*n* = 4)	STRIKE2001 (15pmol)	gp100_25-33_^b^ (37.5pmol)	0
			2 (*n* = 4)	STRIKE2001 (15pmol)	pTag- gp100_25-33_ (37.5pmol)	
4	tghCD40	10	1 (*n* = 6)	STRIKE2001 (150pmol)	pTag-OVA_323-339_ (450pmol)	0, 5
In-vivo tumor growth studies						
1	tgCD40 (tumor cell line: TC-1)	162	1 (*n* = 8)	STRIKE2001 (150pmol)	pTag-HPV-16 E7_44-62_ (450pmol)	5, 10, 15
2	tgCD40 (tumor cell line: TC-1)	173	2 (*n* = 8)	STRIKE2001 (250pmol)	pTag-HPV-16 E7_44-62_ (750pmol)	5, 10
			3 (*n* = 8)	STRIKE2001 (250pmol)	truncated pTag-HPV-16 E7_44-62_^b^ (750pmol)	
3	tgCD40 (tumor cell line: TC-1)	300	1 (*n* = 13)	STRIKE2001 (250pmol)	pTag-HPV-16 E7_44-62_ (750pmol)	5, 12,19
4	tgCD40 (tumor cell line: MC38)	551	1 (*n* = 8)	STRIKE2001 (150pmol)	pTag-ADPGK (450pmol)	6, 10, 14, 22
			2 (*n* = 8)	STRIKE2001 (150pmol)	Truncated pTag-ADPGK^b^ (450pmol)	
			3 (*n* = 8)	STRIKE200 (150pmol)	-	
5^a^	tgCD40 (tumor cell line: MC38)	779	1 (*n* = 12)	STRIKE2001 (150pmol)	pTag-ADPGK (450pmol)	6, 10, 14
			2 (*n* = 12)	STRIKE2001 (150pmol)	pTag-KRAS_G12V (450pmol)	
6^a^	tgCD40 (tumor cell line: MC38)	860	1 (*n* = 10)	STRIKE2001 (150pmol)	pTag-ADPGK (450pmol)	6, 10, 14
			2 (*n* = 10)	STRIKE2001 (150pmol)	truncated pTag-ADPGK^b^ (450pmol)	
			3 (*n* = 10)	STRIKE2001 (150pmol)	—	

^a^In all experiments, the BiAb/pTag-peptide mixture was administered in a ratio of 1:3, except for experiments 5 and 6, where all mice received an antibody dose of 450 pmol and a peptide dose of 450 pmol (1:1) on day 10. ^b^These peptides carry the same sequence as the corresponding pTag-peptide, except for having a truncated pTag that lacks the ability to bind the bispecific antibody.^c^ BI10 BiAb has the 1150 anti-CD40 backbone with the anti-pTag scFv and BI17 BiAb has the same backbone but with the anti-fTag scFv, we refer the reader to the previous publication^17^ for details on BI10 and BI17 BiAbs. ^d^Number of observations included in the analysis, with vehicle control groups also accounted for.

Three in vivo PK studies were conducted. The first-generation Bi10 antibody was evaluated in the first PK study, whereas later studies used the second-generation STRIKE2001 antibody. Different injection routes were also evaluated because they influence immune activation, systemic exposure, and clinical translatability. Hock injection is a SC route known to be highly effective immunization method in mice, while s.c. flank injection and i.v. administrations were designed to mimic the human administration routes of biologics. In the first PK study, C57BL/6 mice received s.c. Bi10:18mer pTag- gp10025-33 in the hock, with plasma and lymph node (i.e., inguinal and popliteal lymph nodes) samples collected. In the second PK study, human CD40 transgenic mice (tghCD40) received STRIKE2001:pTag-KRAS_G12D via s.c. (flank and hock) and i.v. routes, while the third study used only s.c. flank administration for STRIKE2001:pTag-KRAS_G12V. The second study also included s.c. administration of pTag-KRAS_G12D alone, and in the third study, one group received STRIKE2001 alone (s.c. and i.v.). In these latter two studies, only plasma samples were collected. In all studies, the total BiAb and pTag-peptide (i.e., either in a free or as a conjugate) concentrations were measured.

In the first PK study, plasma samples and draining popliteal and inguinal lymph nodes (i.e., one plasma sample and one each lymph node sample per animal) were collected at 0.5, 1.5, 4, and 8 h, with an additional plasma sample at 24 h. The samples were processed by tryptic digestion^1^, and the antibody and intact peptide were measured using LC-MS/MS analysis. In the second PK study, plasma samples were collected after 0.08, 0.5, 1, 2, 4, 8, and 24 h. Antibody concentrations were measured by Admescope (Oulu, Finland), and intact peptide concentrations were measured by Recipharm (Uppsala, Sweden). In the third PK study, plasma samples were collected at 0.08, 0.5, 1, 2, 4, 8, 16, 24, 30, and 48 h, and antibody and intact peptide concentrations were analyzed as in the second PK study. In the second and third PK studies, three plasma samples were collected per animal.

To evaluate peptide uptake and release, an in vitro assay was performed in which immature DCs were incubated in duplicates with Bi17:fluorescent tag (fTag)-CMV pp65_495-503_, Bi17, fTag-CMV pp65_495-503_, or phosphate-buffered saline (PBS). The peptide uptake was assessed over time by quantifying DCs with intracellularly released fTag-CMV pp65_495-503_, and those with both extracellularly attached and intracellularly released peptide.

In vivo T-cell expansion studies in tghCD40 mice assessed CD8^+^ T-cell expansion (two studies via IFNγ Fluorospot and one study via flow cytometry) and a fourth study evaluated CD4^+^ T-cell expansion (IL-2 Fluorospot) after s.c. vaccination in the hock. In the second and third studies, PMEL-1-specific immune cells, and in the fourth, OT-II-specific immune cells, were adoptively transferred one day before vaccination. Post-vaccination, inguinal and popliteal lymph nodes and spleens were collected for analysis.

The antitumor efficacy was investigated in tumor growth experiments using two tumor models, TC-1 and MC38. The TC-1 cell line (from Johns Hopkins) has been established by the introduction of the human oncogenes E6 and E7 (i.e., viral antigens). The selected peptide is a well-established E7-derived MHC class I epitope known to induce anti-tumor responses. For the TC-1 tumor experiments, 5 × 10^4^ cells in PBS were injected s.c. in the flank (i.e., corresponds to 0.05 mm^3^ at time = 0). The MC38 adenocarcinoma colorectal cell line (a kind gift by Mario P. Colombo lab, Milan) has been established through repeated injections of the carcinogen dimethylhydrazine^42,43^, resulting in a colon adenocarcinoma in C57BL/6 mice. The MHC class I-derived neoantigen used in the MC38 model was identified by Yadav et al. (2014) using whole-exome and transcriptome sequencing combined with mass spectrometry, and later confirmed to have in vivo activity by Hos et al. in 2019 and 2023^42,44,45^. For the MC38 tumor experiments, 3 × 10^5^ cells in PBS were injected s.c. in the flank (i.e., corresponds to 0.3 mm^3^ at time = 0). Tumor size was measured every 2–3 days until reaching 1000 mm^3^ or the humane endpoint, defined as either a weight loss exceeding 10% or the presence of visible wounds.

All animal studies were approved by the Uppsala regional ethical committee (Dnr: C304214/16 (C42/14), Dnr: 5.8.18 02686/2019, and Dnr: 5.8.18-18415/2023). In short, mice were housed in a temperature and humidity-controlled facility under a 12 h light/dark cycle with environmental enrichment provided in the cages. All animals had unlimited access to food and water and were monitored daily for general health. For the experimental studies, female and male mice with varying ages were randomly assigned to experimental groups prior to the start of the experiment to reduce selection bias. Anesthesia was not used. Mice were euthanized by cervical dislocation, and blood and tissue samples were collected postmortem.

### A multiscale semi-mechanistic pharmacokinetic-pharmacodynamic modeling framework

The framework was developed sequentially by assembling sub-models describing the key PK and PD processes and the biological interactions required for vaccine-induced T-cell responses and tumor regression, including the temporal interplay between product components (Fig. 2). Model development followed a data-driven approach, integrating in silico, in vitro, and in vivo data while remaining consistent with established literature. Only a limited number of parameters were fixed a priori from published sources. The sub-models are listed below.

A PK modeling analysis was conducted to characterize the systemic and lymphatic disposition of the BiAb and pTag-peptide. Plasma and lymphatic concentrations from all PK studies were simultaneously analyzed to assess the free and conjugated BiAb and pTag-peptide kinetics. Structural models with a central systemic compartment, with or without a peripheral distribution compartment, were tested. Based on mouse anatomy, the lymphatic drainage was divided in parallel into popliteal and inguinal lymph nodes after s.c. administration in the hock^26^. Both linear and non-linear absorption and elimination kinetics were evaluated. The binding kinetics between the BiAb and pTag-peptide were implemented using a target-mediated drug disposition model with two binding sites for peptide, assuming no interaction between the binding sites of the drug^46^. The association (${k}_{{on}}$) and dissociation (${k}_{{off}}$) rate constants were experimentally determined^17,18^ (Table 2).

A multistate model for the peptide uptake by antigen-presenting cells characterized the dynamic states of the APCs (i.e., DCs) in response to the BiAb:pTag-peptide conjugate or its free forms. These states included extracellularly attached and intracellularly released peptides. The transitions between the states were described using first-order rates. The states were defined based on the observations described in the in vitro analyses. Transition delays were evaluated using transit compartments, a modeling construct representing a delay between an upstream event and a downstream response by routing signal through intermediate, unobserved compartments with estimated transfer rates. Due to the lack of experimental data to characterize the timing between the peptide release and its presentation by APCs, this process was assumed to occur instantaneously upon intracellular release.

Within the injection site draining lymph nodes, the activation, proliferation, and differentiation of CD8^+^ and CD4^+^ T-cells in response to peptides presented on the surface of APCs by MHC I and II molecules, respectively, were characterized by a T-cell dynamics model. Two subtypes of T-cells were included in the analysis: transitional effector memory T-cells with a short half-life and central memory T-cells with a long half-life. Both subtypes were assumed to evolve sequentially^47^.

The tumor growth dynamics model characterized tumor volume changes in the TC-1 and MC38 mouse models. Unperturbed tumor growth was described from the control group data by evaluating linear, exponential, exponential-linear, Gompertz, and logistic growth models^48^. The impact of the vaccine peptide-specific T-cells, BiAb, and peptide concentrations (i.e., either pTag-peptide or truncated pTag-peptide) on the tumor reduction, and their interaction, was investigated. Linear and non-linear (i.e., Emax, sigmoidal Emax) relationships and effect delays were evaluated. Tumor measurements of ≤ 4.2 mm^3^, regarded to be below the lower limit of quantification, were treated as censored data, using the M3 method^49^. IAV was quantified. The effect of sex and age was assessed.

### Model applications

Using the final PKPD modeling framework, 1000 virtual mice (i.e., each weighing 20 g) were generated to evaluate the impact of modifying the binding affinity between the pTag and anti-pTag, by adjusting the original ${k}_{{off}}$ 4-fold higher or one-quarter lower, on peptide kinetics, T-cell expansion, and tumor response. The BiAb/p-Tag-peptide mixture, BiAb alone, and BiAb/truncated p-Tag-peptide mixture were evaluated per the original study schedule (Table 3).

### Model development and evaluation

Data analysis and simulations were performed using the nonlinear mixed effect modeling software NONMEM version 7.5.1, executed through Perl-speaks-NONMEM (PsN) version 5.2.6^50,51^. The first-order conditional estimation method with interaction (FOCEI) was used for PK, APC peptide uptake, and T-cell dynamics models, and stochastic approximation expectation maximization (SAEM) with Laplacian extension was applied for the TGD model. A sequential modeling approach was adopted for the development of the sub-models. Simulation-based evaluations assessed model performance.

The parameters’ uncertainties were obtained in NONMEM. R version 4.1.1^52^ was used for data management, model diagnostics, graphical visualization, and evaluations. The selection of the final model was based on the following criteria: (i) OFV (i.e., −2∙log-likelihood) using the likelihood ratio test to compare two nested models such that an OFV decrease of 3.84 was considered statistically significant for adding one extra parameter (df = 1, *α* = 0.05), (ii) precision of the parameter estimates, and (iii) diagnostic plots.

## Supplementary information

Supplementary Material

Supplementary Software File

## Data Availability

The datasets used in this paper came in part from previous publications (Eltahir M, Laurén I, Lord M, et al. An Adaptable Antibody-Based Platform for Flexible Synthetic Peptide Delivery Built on Agonistic CD40 Antibodies. Advanced Therapeutics. 2022;5(7):2200008. doi:10.1002/adtp.202200008, and Mebrahtu A, Laurén I, Veerman R, et al. A bispecific CD40 agonistic antibody allowing for antibody-peptide conjugate formation to enable cancer-specific peptide delivery, resulting in improved T Cell proliferation and anti-tumor immunity in mice. Nat Commun. 2024;15(1):9542. doi:10.1038/s41467-024-53839-5) as well as unpublished data sets, which will be made available in a public repository.
