# Supplementary material for: Evaluating neoantigen-vaccine responses through mechanistic and model-based frameworks
Source: NPJ Precis Oncol. 2026 Jun 24;10:245. doi: 10.1038/s41698-026-01579-8 (PMC13294367; doi:10.1038/s41698-026-01579-8)
Supplement: Supplementary file 1 — Supplementary Material [file 41698_2026_1579_MOESM1_ESM.pdf]

Evaluating Neoantigen-Vaccine Responses through Mechanistic and Model-Based Frameworks

Eman I. K. Ibrahim[1], Ida Laurén[1], Rosanne E. Veerman[3], Siv Jönsson[1], A. Jimmy Ytterberg[2], Annika Lindqvist[2], Sara M. Mangsbo[1,3] and Lena E. Friberg[1]

[1] Department of Pharmacy, Uppsala University, Uppsala, Sweden

[2] Department of Pharmacy, SciLifeLab Drug Discovery and Development Platform, Uppsala University, Uppsala, Sweden

[3] Strike Pharma AB, Lund, Sweden

Supplementary software file

This file provides the model code used for parameter estimation associated with this study.

Supplementary material

1- Pharmacokinetic model

The PK model was composed of the following components: 1) site of s.c. injection in the right flank, 2) site of s.c. injection in the hock, 3) hock injection draining lymph nodes, and 4) plasma.

Site of subcutaneous injection in the right flank

The injection site was initialized with a fully saturated BiAb with pTag-peptide ($BiAb{:}2Ag_{sc}$) (i.e., 1:2), together with the free excess pTag-peptide, in case of 1:3 preparations. For 1:1 mixture, the injection site was initialized with the partially saturated BiAb with one pTag-peptide ($BiAb{:}Ag_{sc}$). The absorption of the free and conjugated BiAb to pTag-peptide into the central compartment were characterized by Michaelis–Menten (MM_{sc}) processes with an estimated maximum absorption capacity of total BiAb ($V_{max,sc}$). The parameter $K_{m,sc}$ described the concentration at which the absorption rate was half-maximal. The free BiAb ($BiAb_{sc}$) was absorbed through an additional first-order rate constant ($k_{sc,BiAb}$) into the central compartment. $k_{sc,Ag}$ represents the first-order absorption rate constant of the free peptide (Ag_{sc}). $BiAb{:}Ag_{sc}$ and $BiAb{:}2Ag_{sc}$ were degraded via a first-order rate constant ($k_{deg,BiAb:Ag}$). See Table S2 for the remaining parameters' descriptions and Equations 1-5.

$$MM_{sc} = \frac{V_{max,sc}}{Km_{sc} + BiAb_{sc} + BiAb{:}Ag_{sc} + BiAb{:}2Ag_{sc}} \quad (1)$$

$$\frac{d\,(BiAb_{sc})}{dt} = -\left(\frac{k_{on}}{V_{inj}}\right)\cdot 2\cdot BiAb_{sc}\cdot Ag_{sc} + k_{off}\cdot BiAb{:}Ag_{sc} - k_{sc,BiAb}\cdot BiAb_{sc} - MM_{sc}\cdot BiAb_{sc} \quad (2)$$

$$\frac{d\,(Ag_{sc})}{dt} = -\left(\frac{k_{on}}{V_{inj}}\right)\cdot 2\cdot BiAb_{sc}\cdot Ag_{sc} - \left(\frac{k_{on}}{V_{inj}}\right)\cdot BiAb{:}Ag_{sc}\cdot Ag_{sc} + k_{off}\cdot BiAb{:}Ag_{sc} + k_{off}\cdot 2\cdot BiAb{:}2Ag_{sc} - k_{sc,Ag}\cdot Ag_{sc} \quad (3)$$

$$\frac{d\,(BiAb{:}Ag_{sc})}{dt} = \left(\frac{k_{on}}{V_{inj}}\right)\cdot 2\cdot BiAb_{sc}\cdot Ag_{sc} - k_{off}\cdot BiAb{:}Ag_{sc} - \left(\frac{k_{on}}{V_{inj}}\right)\cdot BiAb{:}Ag_{sc}\cdot Ag_{sc} + k_{off}\cdot 2\cdot BiAb{:}2Ag_{sc} - MM_{sc}\cdot BiAb{:}Ag_{sc} - k_{deg,BiAb:Ag}\cdot BiAb{:}Ag_{sc} \quad (4)$$

$$\frac{d\,(BiAb{:}2Ag_{sc})}{dt} = \left(\frac{k_{on}}{V_{inj}}\right)\cdot BiAb{:}Ag_{sc}\cdot Ag_{sc} - k_{off}\cdot 2\cdot BiAb{:}2Ag_{sc} - MM_{SC}\cdot BiAb{:}2Ag_{sc} - k_{deg,BiAb:Ag}\cdot BiAb{:}2Ag_{sc} \quad (5)$$

Site of subcutaneous injection in the hock

The s.c. injection in the hock was initiated similarly to the s.c. injection in the right flank. The free ($BiAb_{hc,y}$) and conjugated BiAb to pTag-peptide ($BiAb\!:\!Ag_{hc,y}$ and $BiAb\!:\!2Ag_{hc,y}$) were absorbed through a first-order rate constant ($k_{BiAb,aff,y}$) into the popliteal and inguinal lymph nodes, denoted by index y. $k_{Ag,aff,y}$ depicts the first-order rate constant of absorption of the free peptide ($Ag_{hc,y}$). $BiAb\!:\!Ag_{hc,y}$ and $BiAb\!:\!2Ag_{hc,y}$ were degraded via a first-order rate constant ($k_{deg,BiAb:Ag}$). See Table S2 for the remaining parameters' descriptions and Equations 6-9.

$$\frac{d\left(BiAb_{hc,y}\right)}{dt} = -\left(\frac{k_{on}}{frc_y \cdot V_{inj}}\right) \cdot 2 * BiAb_{hc,y} \cdot Ag_{hc,y} + k_{off} \cdot BiAb\!:\!Ag_{hc,y} - k_{aff,BiAb} \cdot BiAb_{hc,y} \quad (6)$$

$$\frac{d\left(Ag_{hc,y}\right)}{dt} = -\left(\frac{k_{on}}{frc_y \cdot V_{inj}}\right) \cdot 2 \cdot BiAb_{hc,y} \cdot Ag_{hc,y} - \left(\frac{k_{on}}{frc_y \cdot V_{inj}}\right) \cdot BiAb\!:\!Ag_{hc,y} \cdot Ag_{hc,y} + k_{off} \cdot BiAb\!:\!Ag_{hc,y} + k_{off} \cdot 2 \cdot BiAb\!:\!2Ag_{hc,y} - k_{aff,Ag} \cdot Ag_{hc,y} \quad (7)$$

$$\frac{d\left(BiAb\!:\!Ag_{hc,y}\right)}{dt} = \left(\frac{k_{on}}{frc_y \cdot V_{inj}}\right) \cdot 2 \cdot BiAb_{hc,y} \cdot Ag_{hc,y} - k_{off} \cdot BiAb\!:\!Ag_{hc,y} - \left(\frac{k_{on}}{frc_y \cdot V_{inj}}\right) * BiAb\!:\!Ag_{hc,y} \cdot Ag_{hc,y} + k_{off} \cdot 2 \cdot BiAb\!:\!2Ag_{hc,y} - k_{aff,BiAb} \cdot BiAb\!:\!Ag_{hc,y} - k_{deg,BiAb:Ag} \cdot BiAb\!:\!Ag_{hc,y} \quad (8)$$

$$\frac{d\left(BiAb\!:\!2Ag_{hc,y}\right)}{dt} = \left(\frac{k_{on}}{frc_y \cdot V_{inj}}\right) \cdot BiAb\!:\!Ag_{hc,y} \cdot Ag_{hc,y} - k_{off} \cdot 2 \cdot BiAb\!:\!2Ag_{hc,y} - k_{aff,BiAb} \cdot BiAb\!:\!2Ag_{hc,y} - k_{deg,BiAb:Ag} \cdot BiAb\!:\!2Ag_{hc,y} \quad (9)$$

Hock injection draining lymph nodes

Following s.c. injection in the hock, four distinct entities; $BiAb_{hc,y}$, $Ag_{hc,y}$, $BiAb\!:\!Ag_{hc,y}$, and $BiAb\!:\!2Ag_{hc,y}$, were allowed to enter the popliteal and inguinal lymph nodes through the afferent lymphatics. From there, they were absorbed into the central compartment via the efferent lymphatics with a rate described by a first-order rate constant k_{eff}. See Table S2 for the remaining parameters' descriptions and Equations 10-13.

$$\frac{d\left(BiAb_{ln,y}\right)}{dt} = -\left(\frac{k_{on}}{V_y}\right) \cdot 2 \cdot BiAb_{ln,y} \cdot Ag_{ln,y} + k_{off} \cdot BiAb\!:\!Ag_{ln,y} + k_{aff,BiAb} \cdot BiAb_{hc,y} - k_{eff} \cdot BiAb_{ln,y} \quad (10)$$

$$\frac{d\left(Ag_{ln,y}\right)}{dt} = -\left(\frac{k_{on}}{V_y}\right) \cdot 2 \cdot BiAb_{ln,y} \cdot Ag_{ln,y} - \left(\frac{k_{on}}{V_y}\right) \cdot BiAb\!:\!Ag_{ln,y} \cdot Ag_{ln,y} + k_{off} \cdot BiAb\!:\!Ag_{ln,y} + k_{off} \cdot 2 \cdot BiAb\!:\!2Ag_{ln,y} + k_{aff,Ag} \cdot Ag_{hc,y} - k_{eff} \cdot Ag_{ln,y} \quad (11)$$

$$\frac{d\left(BiAb\!:\!Ag_{ln,y}\right)}{dt} = \left(\frac{k_{on}}{V_y}\right) \cdot 2 \cdot BiAb_{ln,y} \cdot Ag_{ln,y} - k_{off} \cdot BiAb\!:\!Ag_{ln,y} - \left(\frac{k_{on}}{V_y}\right) \cdot BiAb\!:\!Ag_{ln,y} \cdot Ag_{ln,y} + k_{off} \cdot 2 \cdot BiAb\!:\!2Ag_{ln,y} + k_{aff,BiAb} \cdot BiAb\!:\!Ag_{hc,y} - k_{eff} \cdot BiAb\!:\!Ag_{ln,y} \quad (12)$$

$$\frac{d\left(BiAb{:}\,2Ag_{ln,y}\right)}{dt} = \left(\frac{k_{on}}{V_y}\right) \cdot BiAb{:}\,Ag_{ln,y} \cdot Ag_{ln,y} - k_{off} \cdot 2 \cdot BiAb{:}\,2Ag_{ln,y} + k_{aff,BiAb} \cdot BiAb{:}\,2Ag_{hc,y} - k_{eff} \cdot BiAb{:}\,2Ag_{ln,y} \quad (13)$$

Plasma (central compartment)

The four entities, $BiAb_{plasma}$, $BiAb{:}\,Ag_{plasma}$, $BiAb{:}\,2Ag_{plasma}$, and Ag_{plasma} were eliminated via the first-order rate constants $k_{el,Ag}$ $(\frac{CL,BiAb}{V,BiAb})$ for the free peptides and $k_{el,BiAb}$ $(\frac{CL,Ag}{V,Ag})$ for the free and the conjugated antibodies. A non-linear elimination was implemented for the total BiAb with maximum capacity (V_{max}) and concentration at which the elimination rate is half-maximal (K_m). See Table S2 for the remaining parameters' descriptions and Equations 14-18.

$$MM_{plasma} = \frac{V_{max}}{K_m + \frac{BiAb_{plasma} + BiAb{:}\,Ag_{plasma} + BiAb{:}\,2Ag_{plasma}}{V_{BiAb,plasma}}} \quad (14)$$

$$\frac{d\left(BiAb_{plasma}\right)}{dt} = -\left(\frac{k_{on}}{V_{BiAb,plasma}}\right) \cdot 2 \cdot BiAb_{plasma} \cdot Ag_{plasma} + k_{off} \cdot BiAb{:}\,Ag_{plasma} + \sum_y k_{eff} \cdot BiAb_{ln,y} + k_{sc,Ab} \cdot BiAb_{sc} + MM_{sc} \cdot BiAb_{sc} - MM_{plasma} \cdot \left(\frac{BiAb_{plasma}}{V_{BiAb,plasma}}\right) - k_{el,BiAb} \cdot BiAb_{plasma} \quad (15)$$

$$\frac{d\left(Ag_{plasma}\right)}{dt} = -\left(\frac{k_{on}}{V_{BiAb,plasma}}\right) \cdot 2 \cdot BiAb_{plasma} \cdot Ag_{plasma} - \left(\frac{k_{on}}{V_{BiAb,plasma}}\right) * BiAb{:}\,Ag_{plasma} \cdot Ag_{plasma} + k_{off} \cdot BiAb{:}\,Ag_{plasma} + k_{off} \cdot 2 \cdot BiAb{:}\,2Ag_{plasma} + \sum_y k_{eff} \cdot Ag_{ln,y} + k_{sc,Ag} \cdot Ag_{sc} - k_{el,Ag} \cdot Ag_{plasma} \quad (16)$$

$$\frac{d\left(BiAb{:}\,Ag_{plasma}\right)}{dt} = \left(\frac{k_{on}}{V_{BiAb,plasma}}\right) \cdot 2 \cdot BiAb_{plasma} \cdot Ag_{plasma} - k_{off} \cdot BiAb{:}\,Ag_{plasma} - \left(\frac{k_{on}}{V_{BiAb,plasma}}\right) \cdot BiAb{:}\,Ag_{plasma} \cdot Ag_{plasma} + k_{off} \cdot 2 \cdot BiAb{:}\,2Ag_{plasma} + \sum_y k_{eff} \cdot BiAb{:}\,Ag_{ln,y} + MM_{sc} \cdot BiAb{:}\,Ag_{sc} - MM_{plasma} \cdot \left(\frac{BiAb{:}\,Ag_{plasma}}{V_{BiAb,plasma}}\right) - k_{el,BiAb} \cdot BiAb{:}\,Ag_{plasma} \quad (17)$$

$$\frac{d\left(BiAb{:}\,2Ag_{plasma}\right)}{dt} = \left(\frac{k_{on}}{V_{BiAb,plasma}}\right) \cdot BiAb{:}\,Ag_{plasma} \cdot Ag_{plasma} - k_{off} * 2 \cdot BiAb{:}\,2Ag_{plasma} + \sum_y k_{eff} \cdot BiAb{:}\,2Ag_{ln,y} + MM_{sc} \cdot BiAb{:}\,2Ag_{sc} - MM_{plasma} \cdot \left(\frac{BiAb{:}\,2Ag_{plasma}}{V_{BiAb,plasma}}\right) - k_{el,BiAb} \cdot BiAb{:}\,2Ag_{plasma} \quad (18)$$

2- Model for the uptake of peptide by antigen-presenting cells

The model consisted of nine compartments (Figure S1) accounting for four different dendritic cell states: 1) free dendritic state, 2) dendritic cell with BiAb:pTag-peptide bound extracellularly (i.e., to CD40 receptors), 3) dendritic cell with free pTag-peptide attached extracellularly, and 4) dendritic cell with free pTag-peptide released intracellularly. At time = 0, state one was initialized by 100,000 cells while all other states were set to zero. State 2 was represented by the sum of the cells in six transit compartments, accounting for the delay before pTag-peptide in the conjugate form is internalized and released intracellularly. The total count of viable dendritic cells was calculated as the sum of all dendritic cell states. The count of fluorescent dendritic cells as a result of both extracellularly and intracellularly released/free pTag peptide in the unquenched analysis was calculated as the sum of states three and four, while the count, as a result of intracellularly released/free pTag peptide in the quenched analysis, was represented by state 4 only.

In the group of dendritic cells that were incubated with a BiAb/pTag-peptide mixture with a ratio of 1:3, the fraction of free pTag-peptide (frc_{free}) was estimated to be 20 %, while in the group of cells treated with pTag-peptide only, it was fixed to 1. A correction factor ($corr$) was estimated to adjust for the control groups' low fluorescent cell counts observed during the flow cytometry analysis. Potential loss of cells due to adherence on the wall of the wells, the parameters F_{unq} and F_q, for unquenched and quenched analysis, respectively, were estimated to account for the fractions of the initial dendritic cells that were detected during the flow cytometry analysis.

Of note, the observed increase in the fluorescent dendritic cells in the quenched analysis over time in the pTag-peptide alone group was non-physiological, but due to the closed system under the *in vitro* experiment settings (see observed data in Figure S4). However, this was not the case in the *in vivo* experiments in which pTag-peptide was rapidly cleared after being injected.

In the simulation-based evaluations (Figure S4), the median of the observations is within the 90% confidence intervals of the predicted medians using the model-estimated parameters, which reflects a good predictive ability of the model. The final model parameters and their uncertainties are presented in Table S3.

3- T-cell dynamics model

At baseline, the vaccine peptide-specific naïve T-cells ($NT_{y,0}$) were calculated based on an estimated fraction (frc_{CD}) of the total immune cells per lymph node ($Immune_y$).

In IFN-γ and IL-2 Fluorospot analysis, spot-forming units (SFU) represent the number of vaccine peptide-specific spots, where each spot corresponds to a single peptide-specific activated T-cell, within a fixed total pool of cells (i.e., experiment-specific). The differentiation between CD8 and CD4 responses in the FluoroSpot assay was based on the use of peptide stimuli specific to each T-cell subset, in accordance with the model used. The equations below describe the computations performed during modelling analysis for SFU derivation;

$$SFU = \frac{(\sum_y NT_y + \sum_y TEM_{CDi,y} + \sum_y CM_{CDi,y}) \cdot total\ pool\ of\ cells}{\sum_y Immune_y} \quad (19)$$

While for flow cytometry;

$$\%\ peptide\ specific\ divided\ Tcells_y = \frac{(TEM_{CDi,y} + CM_{CDi,y}) \cdot 100}{Immune_y \cdot frc_{Tcell,exp4}} \quad (20)$$

4- Supplementary tables

Table S1: *Parameter estimates and their uncertainty (relative standard error, RSE) for the pharmacokinetic model.*

Parameters	Description	Value	RSE (%)
$MTT_{aff,BiAb}\ (h)$	mean transit time from the subcutaneous injection site in the hock to the draining lymph nodes through the afferent lymphatics of free and conjugated BiAb	6.09	10
$MTT_{aff,Ag}\ (h)$	mean transit time from the subcutaneous injection site in the hock to the draining lymph nodes through the afferent lymphatics of free Ag	0.249	19
$MTT_{eff}\ (h)$	mean transit time from the draining lymph nodes to the central compartment through efferent lymphatics	0.0121	15
frc_{pop}	Fraction of the subcutaneous injection in the hock dose draining into popliteal lymph node	0.567	9.3
$MTT_{sc,BiAb}\ (h)$	mean transit time of free BiAb from the subcutaneous injection site in the right flank to the central compartment	33.7	32
$MTT_{sc,Ag}\ (h)$	mean transit time of free Ag from the subcutaneous injection site in the right flank to the central compartment	0.249	19
$V_{max,sc}\ (pmol \cdot h^{-1})$	maximum absorption capacity of total BiAb	38.1	50
$K_{m,sc}\ (nmol)$	amount of total BiAb at which the absorption rate is half-maximal	0.923	53
$t_{1/2 deg,BiAb}\ (h)$	Half-life of BiAb conjugate degradation at the injection site	30.9	69
$CL_{Bi10}\ (ml \cdot h^{-1})$	BiAb (Bi10) apparent clearance	0.140	21
$CL_{BiAb}\ (ml \cdot h^{-1})$	BiAb (STRIKE2001) clearance	0.0224	6.6
$CL_{Ag,Bi10}\ (ml \cdot h^{-1})$	Ag (conjugated to Bi10) apparent clearance	273	12
$CL_{Ag}\ (ml \cdot h^{-1})$	Ag (conjugated to STRIKE2001) apparent clearance	94.4	32
$V_{BiAb,plasma}\ (ml)$	volume of distribution of BiAb	0.929	6.9
$V_{Ag,plasma}\ (ml)$	apparent volume of distribution of Ag	1.28	8.8
$V_{max}\ (pmol \cdot h^{-1})$	maximum elimination capacity of total BiAb (STRIKE2001)	0.033	20
$K_m\ (pM)$	concentration of total BiAb (STRIKE2001) at which the elimination rate is half-maximal	0.0458	6.1
F_{BiAb}	bioavailability of the injected total BiAb (STRIKE2001)	0.658	7.9
V_{inj}	injection volume	Experiment specific	
V_y	lymph node volume	Experimentally measured variable (animal-specific)	
$RUV_{BiAb,plasma,pk1}\ (\%)$[a]	residual unexplained variability of Bi10 plasma concentrations (PK study 1)	63	12
$RUV_{BiAb,lymph\ node,pk1} (\%)$[a]	residual unexplained variability of Bi10 lymph node concentrations (PK study 1)	76	11

$RUV_{Ag,plasma,pk1}(\%)$[a]	residual unexplained variability of 18mer pTag- gp100$_{25\text{-}33}$ plasma concentrations (PK study 1)	51	24
$RUV_{Ag,lymph\ node,pk1}(\%)$[a]	residual unexplained variability of 18mer pTag- gp100$_{25\text{-}33}$ lymph node concentrations (PK study 1)	66	17
$RUV_{\mathrm{BiAb},plasma,pk2}(\%)$[a]	residual unexplained variability of STRIKE2001 plasma concentrations (PK study 2)	61	10
$RUV_{\mathrm{BiAb},plasma,pk3}(\%)$[a]	residual unexplained variability of STRIKE2001 plasma concentrations (PK study 3)	51	11
$RUV_{Ag,plasma,pk2}(\%)$[a]	residual unexplained variability of pTag-KRAS_G12D plasma concentrations (PK study 2)	46	8.4
$RUV_{Ag,plasma,pk3}(\%)$[a]	residual unexplained variability of pTag-KRAS_G12V plasma concentrations (PK study 3)	75	16

[a] *Additive residual error model on log-transformed data.* $MTT = \frac{1}{k}$, $t_{1/2} = \frac{ln\,(2)}{k}$.

Table S2: *Parameter estimates and their uncertainty (relative standard error, RSE) for the uptake of peptide by antigen-presenting cell model.*

**Parameters**	**Description**	**Value**	**RSE (%)**
$t_{1/2up}$ $(h)^a$	half-life of the transition from state 1 to state 2 and state 3	0.319	32
$MTT_{tr,BiAb:Ag}$ (h)	mean transit time from state 2 to state 4	6.20	19
$t_{1/2APC}$ (h)	half-life of the antigen-presenting cells	20.4	23
frc_{free}	fraction of free pTag-peptide	0.188	37
F_{unq}	fraction of the initial dendritic cells that undergoes the unquenched analysis	0.296	4.9
F_q	fraction of the initial dendritic cells that undergoes the quenched analysis	0.386	2.2
$corr$ $(cells)$	correction factor for the control groups	13.9	11
$RUV_{unq,DC}$ $(\%)^b$	Residual unexplained variability of the total dendritic cell counts in the unquenched analysis	30	19
$RUV_{unq,DC,AG}$ $(\%)^b$	Residual unexplained variability of the fluorescent dendritic cell counts in the unquenched analysis	94	12
$RUV_{q,DC}$ $(\%)^b$	Residual unexplained variability of the total dendritic cell counts in the quenched analysis	12	14
$RUV_{q,DC,AG}$ $(\%)^b$	Residual unexplained variability of the fluorescent dendritic cell counts in the quenched analysis	74	13

[a] the half-life of the transition from state 3 to state 4 ($t_{1/2tr,Ag}$) is equal to $t_{1/2up}$. [b] Additive residual error model on log-transformed data. $MTT = \frac{N}{k}$, $t_{1/2} = \frac{ln\,(2)}{k}$.

Table S3: *Parameter estimates and their uncertainty (relative standard error, RSE) for the T-cell dynamic model.*

**Parameters**	**Description**	**Value**	**RSE (%)**
$frc_{CD8,endo}$	fraction of peptide-specific endogenous naïve T-cell out of total immune cells	$5.09\text{x}10^{-6}$	41
$frc_{CD8\&4,adoptive}$	fraction of peptide-specific adoptively transferred naïve T-cell out of total immune cells	$231\text{x}10^{-6}$	21
$frc_{CD8,exp4}$	fraction of peptide-specific adoptively transferred naïve T-cell out of total immune cells in study 4	$14.1\text{x}10^{-6}$	24
$frc_{Tcell,exp4}$	fraction of total T-cells out of total immune cells in study 4	$0.9752\text{x}10^{-3}$	21
$\sigma_{NT,CD8}$ $(day^{-1})^a$	naïve CD8 T-cell activation rate constant	0.628	8.7
$\sigma_{NT,CD4}$ $(day^{-1})^a$	naïve CD4 T-cell activation rate constant	0.978	9.6
$\sigma_{50}(molecules)$	number of peptide-MHC molecules required for half-maximal activation, proliferation, and differentiation rates of T-cells	$11.02\text{x}10^{3}$	97
$RUV_{exp1}(\%)^b$	residual unexplained variability (study 1)	90	18
$RUV_{exp2}(\%)^b$	residual unexplained variability (study 2)	50	19
$RUV_{exp3}(\%)^b$	residual unexplained variability (study 3)	59	14
$RUV_{exp4}(\%)^b$	residual unexplained variability (study 4)	43	27

[a] σ_{NT}, proliferation rate constant (k_p), and differentiation rate constant (k_{diff}) were assumed to be the same. [b] Additive residual error model on log-transformed data.

Table S4: *Parameter values obtained from literature and their definitions.*

Parameter	Definition	Value	ref.
$\rho_{naive}\ (day^{-1})$	naïve T-cells turnover rate constant	0.0029	[5]
$\rho_{TEM,CD8}\ (day^{-1})$	transition effector memory $CD8^{+}$ T-cells turnover rate constant	0.044	[5,6]
$\rho_{CM,CD8}\ (day^{-1})$	central memory $CD8^{+}$ T-cells turnover rate constant	0.0044	[5,6]
$\rho_{TEM,CD4}\ (day^{-1})$	transition effector memory $CD4^{+}$ T-cells turn-over rate constant	0.063	[5,6]
$\rho_{CM,CD4}\ (day^{-1})$	central memory T-cells $CD4^{+}$ turnover rate constant	0.0062	[5,6]
frc_{TEM}	fraction of effector T-cells that differentiate into memory T-cells	0.1	[7]
$Immune\ (cells \cdot g^{-1})$	Total immune cell density of lymph nodes	8.5×10^{9}	[8]
$k_{on,MHCI}\ (pM \cdot day^{-1})$	on-rate for T-epitope-MHC-I binding	1.8144×10^{-2}	[5]
$k_{on,MHCII}\ (pM \cdot day^{-1})$	on-rate for T-epitope-MHC-II binding	8.64×10^{-3}	[5]

5- Supplementary figures

Figure S1

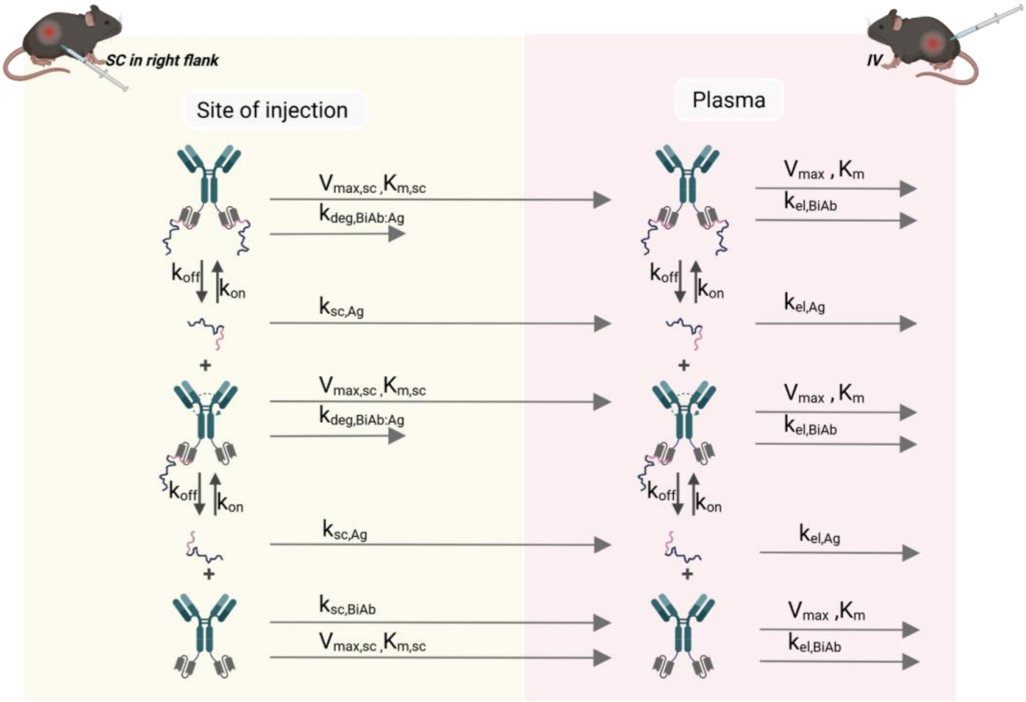

Figure S1: *Schematic representation of the multiscale semi-mechanistic data-driven pharmacokinetic model for the intravenous and subcutaneous in the right flank administration. Abbreviations:* $BiAb$*, bispecific antibody;* Ag*, peptide;* $k_{deg,BiAb:Ag}$*, first-order degradation rate constant of* $BiAb$ *from injection site;* $V_{max,sc}$*, maximum absorption capacity of total* $BiAb$*;* $K_{m,sc}$ *, concentration at which the absorption rate is half-maximal;* $k_{sc,BiAb}$ *and* $k_{sc,Ag}$*, first-order absorption rate constants of free* $BiAb$ *and* Ag *into plasma, respectively;* $k_{el,BiAb}$ *and* $k_{el,Ag}$*, first-order rate elimination constants of* $BiAb$ *and* Ag *from plasma, respectively;* V_{max}*, maximum elimination capacity of total* $BiAb$*;* K_m *, concentration at which the elimination rate is half-maximal. Created in https://BioRender.com.*

Figure S2

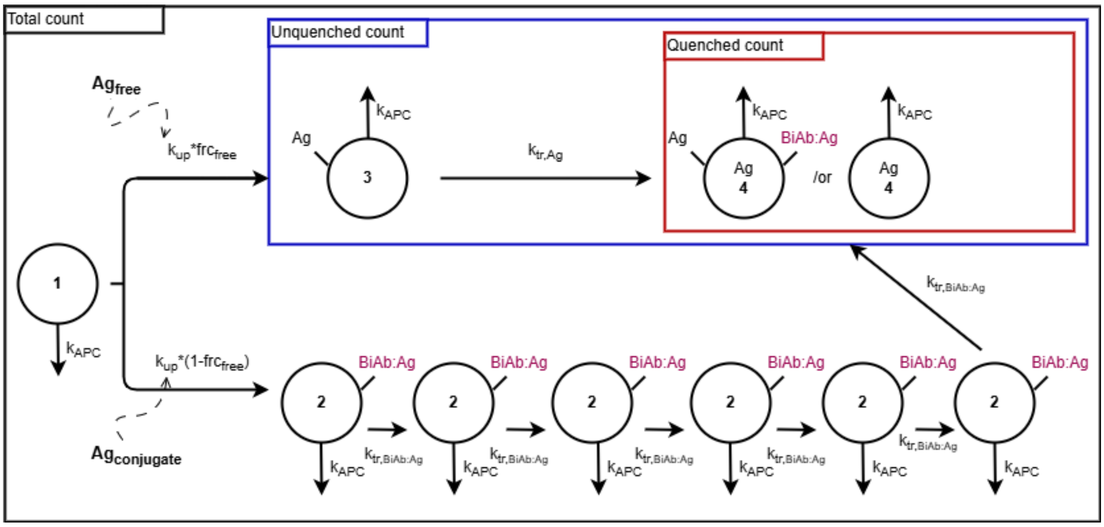

Figure S2 *Schematic representation of the uptake of peptide by antigen-presenting cell model. Black circles illustrate dendritic cells. The black box represents all dendritic cell states accounted for by the total viable count. The blue box represents the count of the fluorescent cells obtained from the unquenched analysis. The red box represents the count of the fluorescent cells obtained from the quenched analysis.*

Figure S3

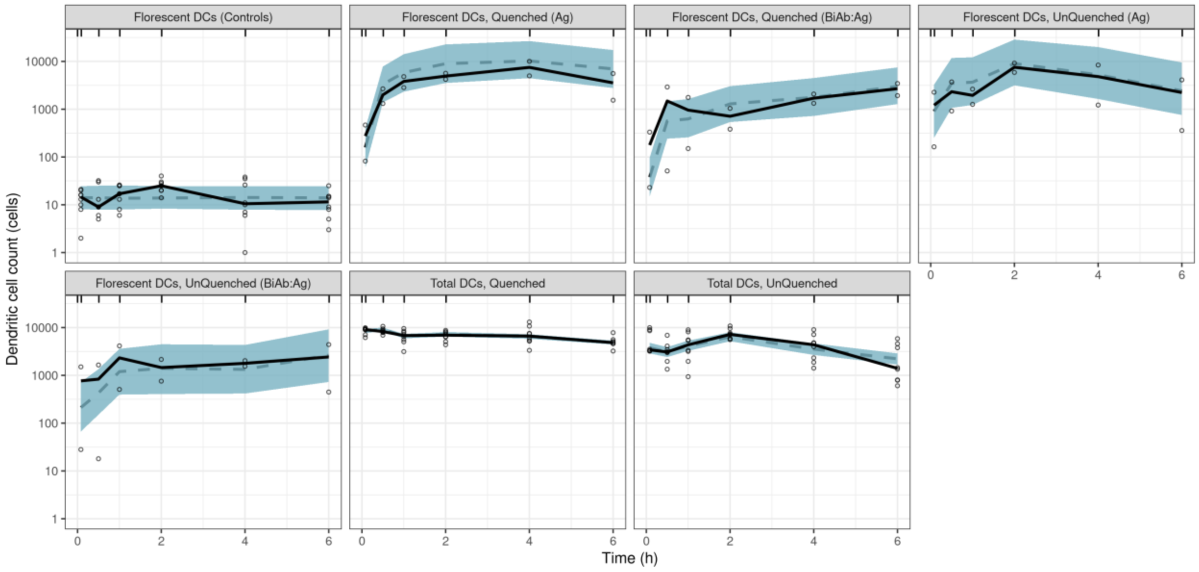

Figure S3: *Simulation-based evaluations of the model for the uptake of peptide by antigen-presenting cells using 1000 simulations. The black dots are the observed cell counts. The solid and dashed black lines are the median of the observed and simulated dendritic cell (DC) count-time profiles, respectively. The blue-shaded areas are the 90% confidence interval of the predicted medians based on the simulated data. Refer to Supplementary Material, section 3, for further details on the analysis.*

Figure S4

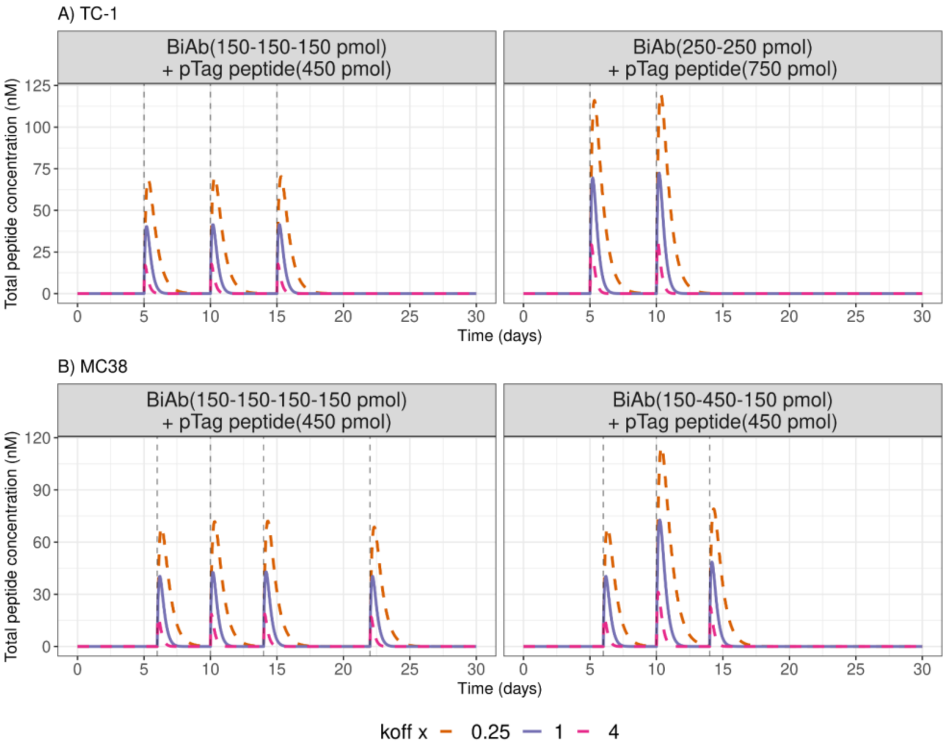

Figure S4: *Model-predicted total peptide concentration-time profiles in plasma in TC-1 (A) and MC38 (B) tumor models, following treatment with BiAb (STRIKE2001) and pTag-peptide (pTag-HPV-16 E7$_{44\text{-}62}$ in TC-1 and pTag-ADPGK in MC38). The vertical dashed lines indicate the dosing times.*

Figure S5

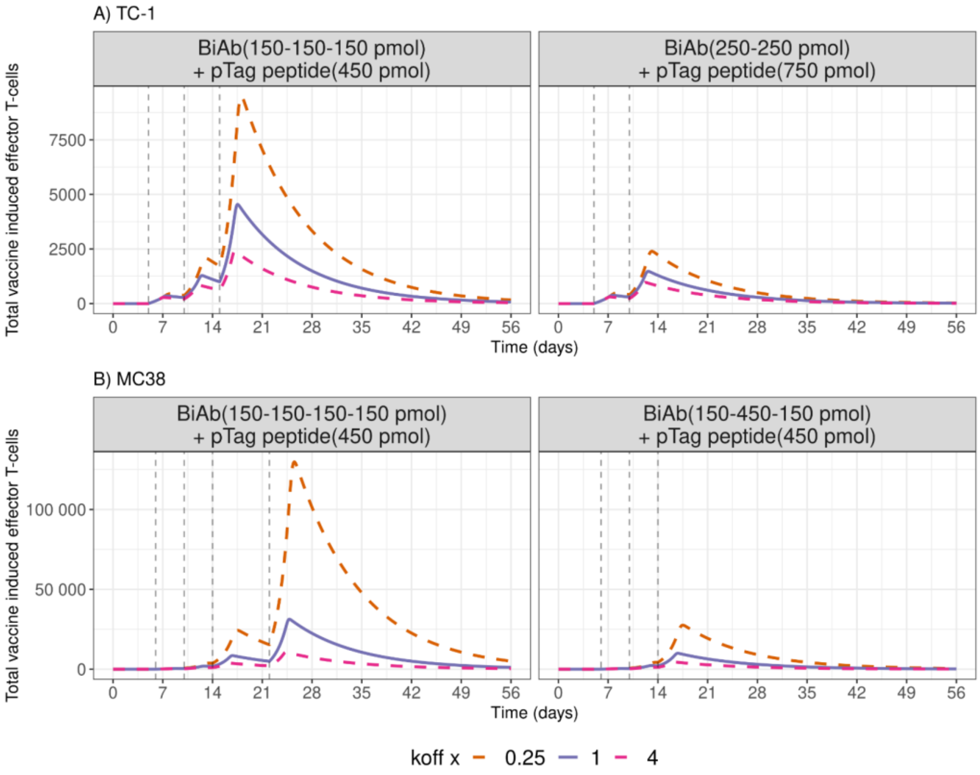

Figure S5: *Model-predicted vaccine-induced effector CD8 T-cell time profiles in TC-1 (A) and MC38 (B) tumor models, following treatment with BiAb (STRIKE2001) and pTag-peptide (pTag-HPV-16 E7$_{44-62}$ in TC-1 and pTag-ADPGK in MC38). The vertical dashed lines indicate the dosing times.*

Figure S6

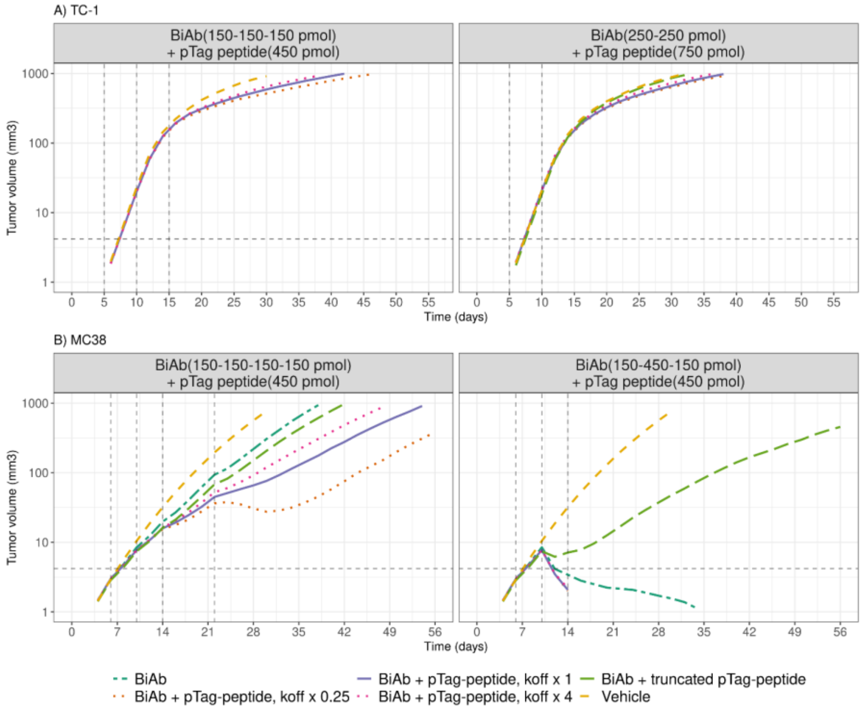

Figure S6: *Model simulations of the tumor volume-time profiles in TC-1 (A) and MC38 (B) tumor models, following treatment with BiAb (STRIKE2001) and pTag-peptide (pTag-HPV-16 E7$_{44-62}$ in TC-1 and pTag-ADPGK in MC38). The colored lines are the simulated median. The vertical dashed lines are the dosing times. The horizontal dashed line is the LLOQ of 4.2 mm^3.*

Figure S7

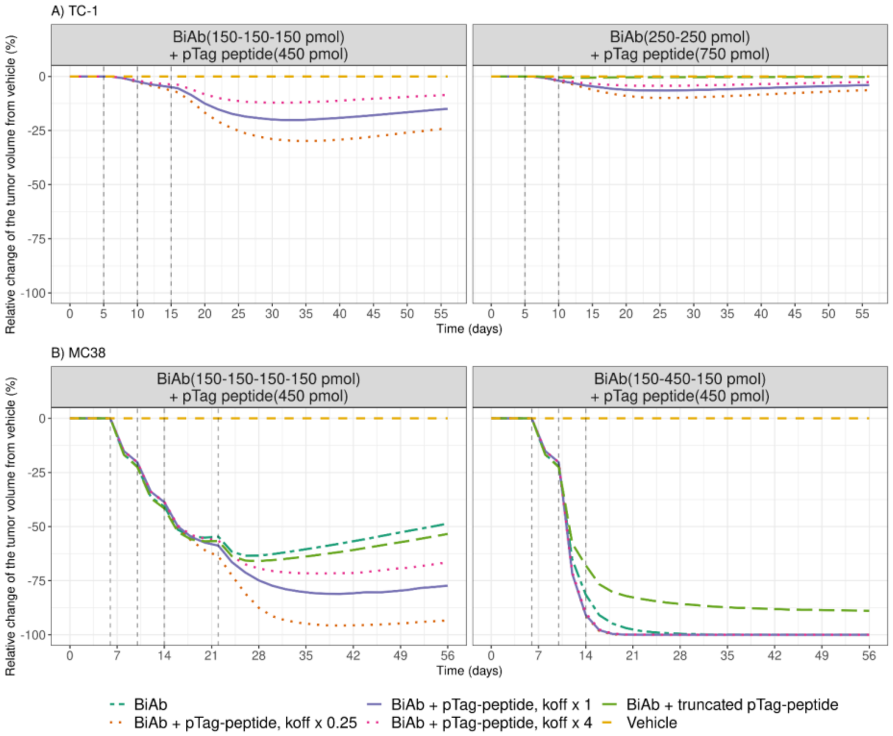

Figure S7: *Model simulations of relative change of tumor volume from vehicle in TC-1 (A) and MC38 (B) tumor models, following treatment with BiAb (STRIKE2001) and pTag-peptide (pTag-HPV-16 E7$_{44-62}$ in TC-1 and pTag-ADPGK in MC38). The colored lines are the simulated medians. The vertical dashed lines are the dosing times.*

Figure S8

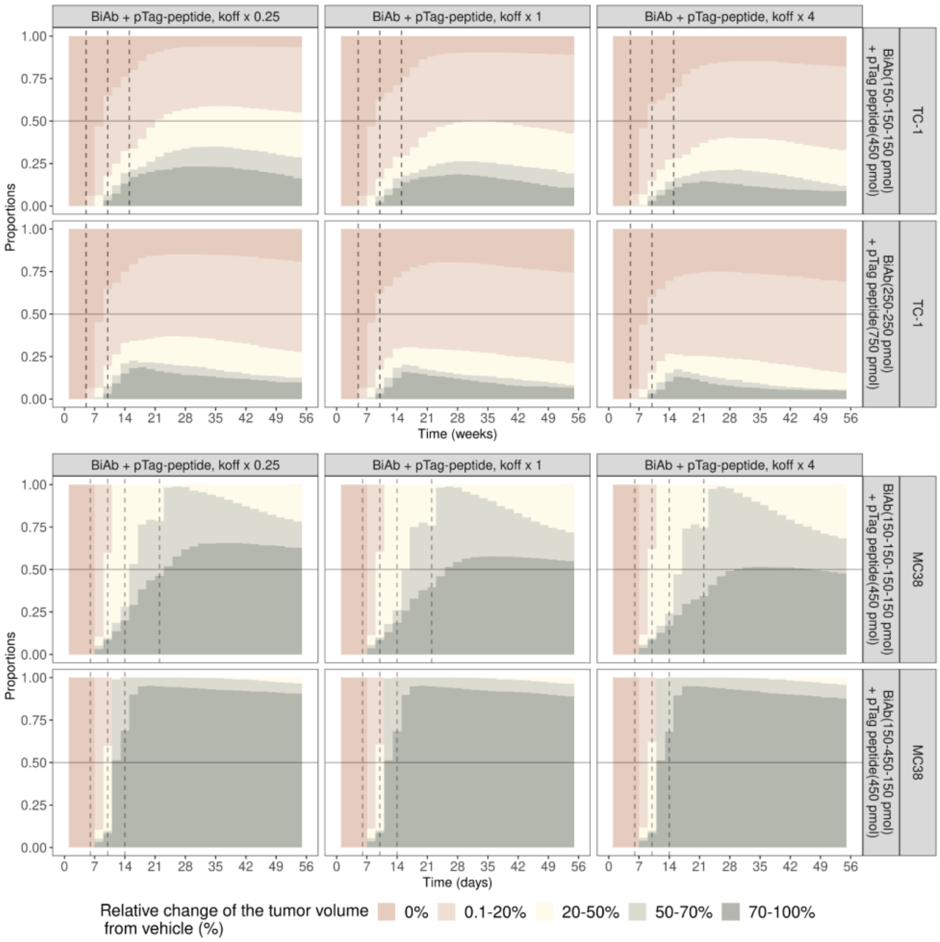

Figure S8: *The model-simulated proportions of responders in TC-1 (upper panel) and MC38 (lower panel) tumor models over time, following treatment with BiAb (STRIKE2001) and pTag-peptide (pTag-HPV-16 E7$_{44\text{-}62}$ in TC-1 and pTag-ADPGK in MC38). The response is determined based on each mouse's tumor growth profile relative to its corresponding vehicle control. The vertical dashed lines indicate the dosing times.*

Figure S9

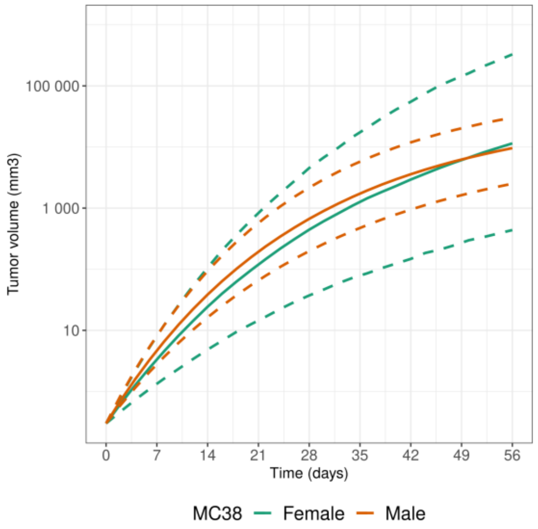

Figure S9: *Model simulations of the tumor volume-time profiles of the MC38 tumor model, stratified by sex, without treatment. The solid lines are the simulated medians. The dashed lines are the 10th and 90th percentiles of the simulation intervals.*

Figure S10

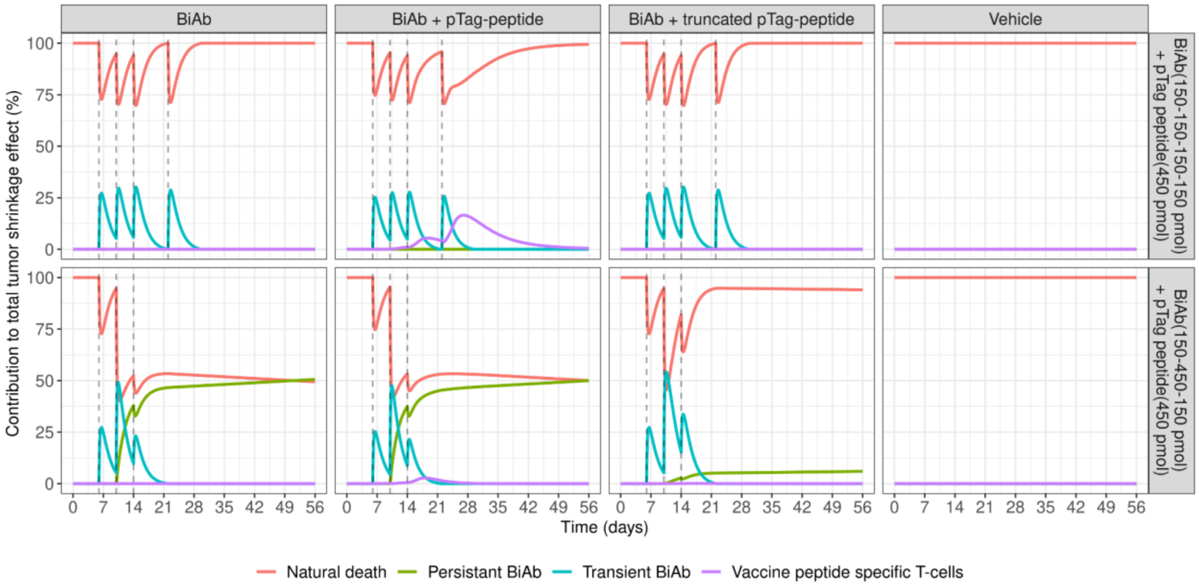

Figure S10: *The model-predicted contributions of the different tumor shrinkage effects to the total in the MC38 tumor model over time, following treatment with BiAb (STRIKE2001) and pTag-peptide (pTag-ADPGK). The vertical dashed lines indicate the dosing times.*

References:

1. R Core Team. R: A Language and Environment for Statistical Computing. Vienna, Austria; 2022. Available from: https://www . R-project.org/.

2. Eltahir M, Laurén I, Lord M, et al. An Adaptable Antibody-Based Platform for Flexible Synthetic Peptide Delivery Built on Agonistic CD40 Antibodies. *Advanced Therapeutics*. 2022;5(7):2200008. doi:10.1002/adtp.202200008

3. Mebrahtu A, Laurén I, Veerman R, et al. A bispecific CD40 agonistic antibody allowing for antibody-peptide conjugate formation to enable cancer-specific peptide delivery, resulting in improved T Cell proliferation and anti-tumor immunity in mice. *Nat Commun*. 2024;15(1):9542. doi:10.1038/s41467-024-53839-5

4. Reynisson B, Alvarez B, Paul S, Peters B, Nielsen M. NetMHCpan-4.1 and NetMHCIIpan-4.0: improved predictions of MHC antigen presentation by concurrent motif deconvolution and integration of MS MHC eluted ligand data. *Nucleic Acids Research*. 2020;48(W1):W449-W454. doi:10.1093/nar/gkaa379

5. Messan MR, Yogurtcu ON, McGill JR, Nukala U, Sauna ZE, Yang H. Mathematical model of a personalized neoantigen cancer vaccine and the human immune system. *PLOS Computational Biology*. 2021;17(9):e1009318. doi:10.1371/journal.pcbi.1009318

6. Hosseini I, Gadkar K, Stefanich E, et al. Mitigating the risk of cytokine release syndrome in a Phase I trial of CD20/CD3 bispecific antibody mosunetuzumab in NHL: impact of translational system modeling. *npj Syst Biol Appl*. 2020;6(1):1-11. doi:10.1038/s41540-020-00145-7

7. Wei SC, Duffy CR, Allison JP. Fundamental Mechanisms of Immune Checkpoint Blockade Therapy. *Cancer Discovery*. 2018;8(9):1069-1086. doi:10.1158/2159-8290.CD-18-0367

8. Sender R, Weiss Y, Navon Y, et al. The total mass, number, and distribution of immune cells in the human body. *Proc Natl Acad Sci U S A*. 120(44):e2308511120. doi:10.1073/pnas.2308511120
